# Salivary Gland Function, Antioxidant Defence and Oxidative Damage in the Saliva of Patients with Breast Cancer: Does the *BRCA1* Mutation Disturb the Salivary Redox Profile?

**DOI:** 10.3390/cancers11101501

**Published:** 2019-10-08

**Authors:** Beata Sawczuk, Mateusz Maciejczyk, Magdalena Sawczuk-Siemieniuk, Renata Posmyk, Anna Zalewska, Halina Car

**Affiliations:** 1 Department of Prosthodontics, Medical University of Bialystok, Sklodowskiej 24a, 15-276 Bialystok, Poland; 2 Department of Hygiene, Epidemiology and Ergonomics, Medical University of Bialystok, Mickiewicza 2C, 15-222 Bialystok, Poland; mateusz.maciejczyk@umb.edu.pl; 3 Department of Orthodontics, Medical University of Bialystok, Waszyngtona 15a, 15-274 Bialystok, Poland; sawczuk.magda@tlen.pl; 4 Department of Perinatology, Medical University of Bialystok, Sklodowskiej 24a, 15-276 Bialystok, Poland; rposmyk@gmail.com; 5 Department of Restorative Dentistry, Medical University of Bialystok, Sklodowskiej 24a, 15-276 Bialystok, Poland; 6 Department of Clinical Pharmacology, Medical University of Bialystok, Waszyngtona 15a, 15-274 Bialystok, Poland; hcar@umb.edu.pl

**Keywords:** breast cancer, saliva, oxidative stress, redox biomarkers, salivary diagnostics

## Abstract

Oxidative stress plays a key role in breast cancer progression. However, little is still known about the relationship between the *BRCA1* mutation, the incidence of breast cancer and oral homeostasis. This is the first study to evaluate the secretory function of salivary glands, biomarkers of redox balance, and oxidative damage to proteins and lipids in the saliva of subjects with the *BRCA1* mutation. Ninety eight women were enrolled in the study and allocated to four groups based on molecular DNA testing: generally healthy patients without the *BRCA1* mutation, patients with breast cancer but without the *BRCA1* mutation, generally healthy patients with the *BRCA1* mutation, and patients with both breast cancer and the *BRCA1* mutation. We demonstrated that saliva from breast cancer patients with the *BRCA1* mutation is characterized by enhanced antioxidant capacity and a higher degree of oxidative damage to proteins and lipids. The *BRCA1* mutation can cause a predisposition to early salivary gland dysfunction, both in patients with breast cancer and in healthy individuals, leading to a decrease in salivary proteins. Using cluster analysis, we showed that salivary peroxidase, advanced glycation end-products (AGE), total antioxidant status (TAS) and malondialdehyde (MDA) may have particular clinical significance in non-invasive diagnostics of breast cancer.

## 1. Introduction

Breast cancer is the most common malignancy in women. The incidence rates of breast cancer increase in women aged between 45 and 54 years and decrease after the menopause [1]. Interestingly, 16–25% of breast cancers are caused by mutations in the *BRCA1* and *BRCA2* genes. *BRCA1* encodes a suppressor protein interacting with other regulatory proteins (e.g., ataxia telangiectasia mutated protein (ATM), ataxia telangiectasia and Rad3-related protein (ATR), p53), oncogenes (e.g., c-Myc, transcription factors E2F1, STK15), proteins involved in DNA repair (e.g., bloom syndrome protein (BLM), MLH1, RAD5) and cell cycle regulators (e.g., MYC, transcription factor JunB, protein coding ATF1) [2]. In this way, *BRCA1* prevents permanent damage to the DNA helix and loss of control over a cell. It has been suggested that *BRCA1* is also involved in the regulation of cell redox homeostasis [3,4]. It is well known that a disturbed balance between the formation and neutralization of reactive oxygen species (ROS) results in structural and functional abnormalities, which is defined as oxidative stress [5]. Increased ROS synthesis leads to oxidative damage to proteins, lipids and DNA, which is a direct cause of the onset and progression of breast cancer and other malignancies. Indeed, disturbances of redox balance and severe oxidative damage have been observed in both serum/plasma as well as tumor in breast cancer patients [6,7,8].

Increasing attention is being drawn to the relationship between general human health and oral health. Recent studies have indicated a relationship between the incidence of periodontal disease and breast cancer. Söder et al. [9] reported a more than two-fold higher incidence of breast cancer in women with periodontal disease compared to those without periodontal disease. Alurfan et al. [10] found reduced salivary pH and total protein concentration in unstimulated saliva from women with breast cancer, while Gornitsky et al. [11] showed significantly higher concentrations of an oxidative DNA damage marker (8-oxo-7-hydrodeoxyguanosine). Considering that oxidative stress is a major phenotypic hallmark in patients with breast cancer [12,13], it can be assumed that in breast cancer patients it is also responsible for pathologies within the oral cavity and salivary glands. It is well known that oxidative stress is one of the main causes of impaired secretory function of the salivary glands, but it is also responsible for changes in the composition of saliva [14,15]. The available literature does not, however, provide any information on the relationship between the *BRCA1* mutation, incidence of breast cancer and salivary antioxidants, oxidative damage and the secretory function of salivary glands [16]. Moreover, very little is still known about the use of salivary biomarkers of oxidative stress in the diagnosis of breast cancer. Therefore, the aim of the present study was to assess the secretory function of salivary glands, selected salivary biomarkers of redox balance, and oxidative damage to proteins and lipids in subjects with breast cancer as compared to patients without breast cancer, positive or not positive for *BRCA1*.

## 2. Material and Methods

### 2.1. Patients

The study was approved by the Local Bioethics Committee of the Medical University of Białystok (1.09.2015 /R-I-002/58/2015). All patients received information on the study protocol and gave written consent to participate in the experiment.

Overall, 98 women (Table 1) treated at the Prosthetics Department of the Medical University of Białystok and the Podlasie Medical Centre, Genetics, were enrolled in the study. Patients were allocated to four groups based on molecular DNA testing and medical history:

- NZ (negative healthy)—generally healthy patients without the *BRCA1* mutation (25 women),
- NC (negative with cancer)—patients with breast cancer but without the *BRCA1* mutation (nine women),
- PZ (positive healthy)—generally healthy patients with the *BRCA1* mutation (22 women),
- PC (positive with cancer)—patients with both breast cancer and the *BRCA1* mutation (20 women).

The control group (NZ, *n* = 25) comprised generally healthy subjects (aged 29–47 years) who had their check-up visits at the Prosthodontics Department of the Medical University of Białystok, Poland. The experimental groups NC and PC comprised patients with grade 3 ductal carcinoma (G3) diagnosed based on a histopathological examination by an experienced pathologist according to World Health Organization (WHO) criteria. The material for tests (blood and saliva) was collected before oncological treatment. To identify the DNA mutation, 10 mL of venous blood was collected into test tubes pre-filled with 10% ethylenodiaminetetraacetic acid (EDTA). DNA was isolated using the ready-made DNeasy Mini Kit column kit (Qiagen, Hilden, Germany). The Ex20- 5382insC, Ex11.17- 4153delA and Ex5- 300T/G mutations of the *BRCA1* gene were evaluated using multiplex PCR and a ready reagent kit (Qiagen, Hilden, Germany), the original composition of the PCR reaction mixture containing primer kits, buffer and synthetic nucleotides) protected by the patent; Department of Genetics, PMU, patent no. PL185597.

We excluded patients with chronic systemic diseases such as: metabolic diseases (obesity, type 1 and type 2 diabetes), cardiovascular diseases (hypertension, ischaemic heart disease, atherosclerosis, arrhythmias and conductivity disorders), autoimmune diseases (scleroderma, rheumatoid arthritis, Sjögren syndrome), gastrointestinal tract diseases, infectious diseases (infection with human immunodeficiency virus (HIV), hepatitis C virus (HCV)), immune disorders, other cancers, and chronic inflammatory diseases. We also excluded pregnant/breastfeeding women, smoking patients, alcohol-dependent women, those chronically taking any medications (including antibiotics, steroids, antihistamines, decongestants, antidepressants, antipsychotics, antihypertensive, anticholinergics and non-steroidal anti-inflammatory drugs), food supplements or vitamins, and those not suffering from active periodontal disease (probing pocket depth (PPD) ≤ 2; bleeding on probing (BOP) < 15) or caries (decayed teeth < 1).

### 2.2. Saliva Collection

The research material was unstimulated mixed saliva collected from patients by the spitting method. Saliva was always collected between 8 am and 10 am, at least 2 hours after a solid or liquid meal other than water. The subjects were instructed to rinse their mouth three times with distilled water and to spit saliva into a sterile Falcon test tube placed in a container with ice. Saliva was sampled for 15 min, and saliva collected within the first minute was discarded [17]. Saliva collection was done by the patient when sitting with the head down, after at least a 5-min adaptation, always in the same isolated room. Sampled saliva was immediately centrifuged (20 min, 4 °C, 3000× *g*), and an antioxidant, butyl-hydroxytoluene (BHT) (Sigma-Aldrich, Hamburg, Germany), was added to the obtained supernatants [18,19]. The supernatants were portioned into smaller 200 μL aliquots and stored at –80 °C for further analysis.

The pH and the buffer capacity of saliva (SBC) were analyzed immediately after the collection of saliva using a Seven Multi pH meter (Mettler Toledo, Greifensee, Switzerland). The pH was measured in a 0.5 mL sample of saliva, and then 2.5 µL (0.1 M) of hydrochloric acid was added. The sample was vigorously mixed and pH was measured again. The buffer capacity was calculated according to the formula: buffer capacity = number of moles of hydrochloric acid added/(v of solution × ΔpH) [20].

### 2.3. Dental Examination

The dentition and oral health of patients were assessed visually by an experienced dentist in a dental surgery using a WHO-621 periodontal probe, a dental probe, and a mouth mirror in the sitting position, under light from a shadowless head lamp. Oral hygiene was assessed using the Pl.I score (Plaque Index). The score was determined by examining the presence of dental plaque on four surfaces of six teeth. It was assumed that scores of the Pl.I index in the range of 0–0.6 indicate good oral hygiene, 0.7–1.8 indicates moderate hygiene, and 1.9–3.0 indicates poor hygiene. The intensity of caries was expressed as the DMFT (decay, missing, filled teeth) index, illustrating the history of caries. This index is the ratio of the sum of teeth with carious lesions, teeth removed for complicated caries, and teeth with fillings to the number of examined teeth [21]. In 30 patients, we assessed the inter-rater agreements between the examiner (B.S.) and another experienced dentist (M.S.). The reliability for the dental indices was >0.96.

### 2.4. Biochemical Tests

#### 2.4.1. Redox Status

Total antioxidant status (TAS) and total oxidant status (TOS) were determined using commercially available diagnostic kits (ImAnOx (TAS/TAC) Kit, Immundiagnostik, Bensheim, Germany, and PerOx (TOS/TOC) Kit, Immundiagnostik, Bensheim, Germany) following the manufacturer’s instructions. The oxidative stress index (OSI) was calculated from the formula: OSI = TOS/TAS × 100 [22].

#### 2.4.2. Salivary Antioxidants

The activity of salivary peroxidase (Px) was determined colorimetrically in a reaction with Ellman’s reagent (5,5’-dithiobis-2-nitrobenzoic acid, DTNB) by measuring Px-dependent changes in the absorbance of the reaction product, 5-thio-2-nitrobenzoic acid, at 412 nm [23]. Catalase activity (CAT) was determined using a method proposed by Aebi et al. [24], by measuring the drop in the absorbance of hydrogen peroxide (H_2_O_2_) at 240 nm. The total phenolic content (pPh) was determined colorimetrically in a reaction with Folin–Ciocalteu’s reagent using the gallic acid calibration curve (gallic acid equivalents, GAE) [25].

#### 2.4.3. Oxidative Damage Products

The concentration of advanced oxidation protein products (AOPP) was measured colorimetrically using a method proposed by Kalousová et al. [26] at 340 nm, and expressed in chloramine units. The fluorescence of advanced glycation end-products (AGE) was measured using a method proposed by Kalousová et al. [26] at an excitation wavelength of 440 nm and an emission wavelength of 350 nm, and expressed in arbitrary fluorescence units (AFU). The concentration of malondialdehyde (MDA) was measured against the blank as thiobarbituric acid reactive substances (TBARS), using a reference curve for 1,1,3,3-tetramethoxypropane [27]. The concentration of 8-isoprostane (8-isop) was measured using enzyme-linked immunosorbent assay (ELISA) and a commercially available kit, 8-isoprostane Cayman Chemical (Ann Arbor, Miami, FL, USA), following the manufacturer’s instructions.

#### 2.4.4. Total Protein (TP)

The concentration of total protein (TP) was measured using the bicinchoninic acid (BCA) assay and a Thermo Scientific PIERCE BCA (Protein Assay Kit, Rockford, IL, USA).

All measurements were done in replicates, and those for TAS, Px and CAT in triplicates. To standardize results, all statistics were converted to 100 mg of total protein.

### 2.5. Statistical Analysis

Obtained data were analyzed using R software, version 2.15.0 (The R Foundation for Statistical Computing, Vienna, Austria, 2012). The normality of distribution for examined parameters was analyzed with the W (Shapiro–Wilk, Miami, FL, USA) test at the significance level of *p* = 0.05. The measurement scales used for parameters were standardized using the scale function from the base package. The standardization of parameters allowed for the comparison of previously non-comparable values of variables. The non-parametric test was used because the analyzed groups differed for their size, and the distribution of variables was non-normal. The significance of intergroup differences was assessed with the post-hoc Dunn’s test. For all tests, the level of significance was adopted at *p* ≤ 0.05.

Spearman”s rank correlation coefficient was used to analyze the relationships between individual quantitative variables. The adopted intervals indicated: 0.2–0.4, weak correlation; 0.4–0.6, moderate correlation; 0.6–0.8, strong correlation; and >0.9, very strong correlation. Differences were statistically significant at *p* < 0.05.

A hierarchical cluster analysis was performed to identify similar groups. The process of grouping was illustrated using the heatmap.2 function from the gplots v. 3.0.1. package (Datanovia, Montpellier, France) The quality of clustering was evaluated using the silhouette function, which determines the similarity of objects within the same cluster.

## 3. Results

Characteristics of analyzed groups are presented in Table 1. Women diagnosed with breast cancer were of reproductive or postmenopausal age. However, there were no statistically significant differences in age between the examined groups. In most patients without the *BRCA1* mutation, cancer was located in the right breast. All patients suffered from the ductal type of breast cancer.

The salivary buffer capacity was highest in healthy women without the *BRCA1* mutation, and much lower in patients with the *BRCA1* mutation and those without the mutation but suffering from breast cancer. There were no significant differences between the groups in the pH of saliva and DMFT and Pl.I scores (Table 2).

**Figure 1:**
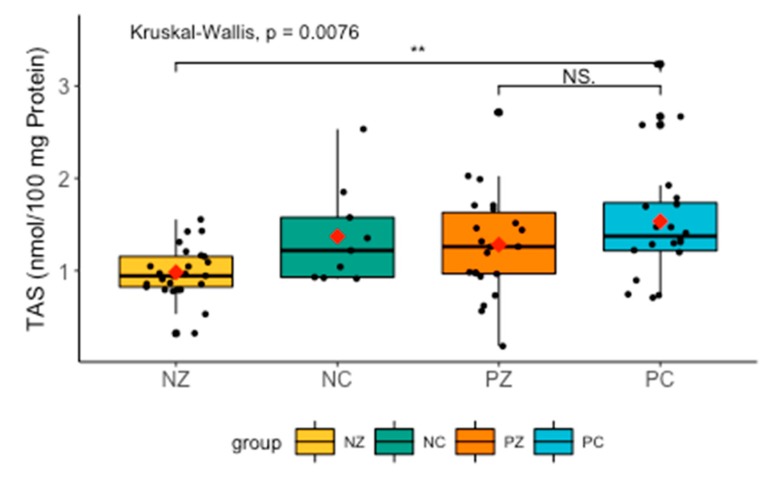
Levels of TAS in the saliva of the examined women. TAS, total antioxidant status; NZ, generally healthy patients without the *BRCA1* mutation; NC, patients with breast cancer but without the *BRCA1* mutation; PZ, generally healthy patients with the *BRCA1* mutation; PC, patients with both breast cancer and the *BRCA1* mutation. ** *p* < 0.01.

TAS levels differed significantly between the groups. TAS tended to be lower in NZ, but higher in patients without the *BRCA1* mutation compared to NZ (H = 11.9263, *p* = 0.0076) (Figure 1).

**Figure 2:**
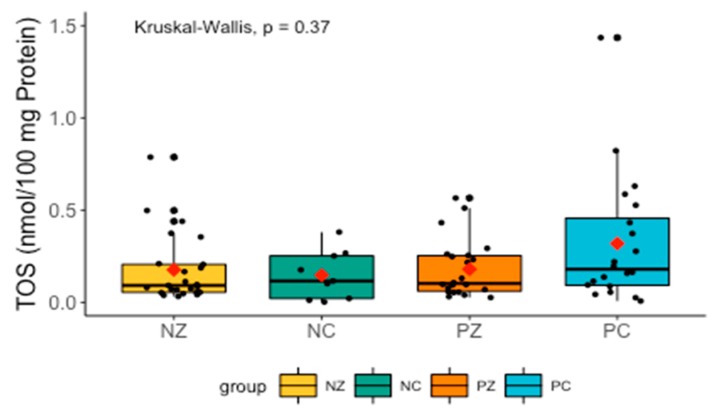
Levels of TOS in the saliva of the examined women. TOS, total oxidant status; NZ, generally healthy patients without the *BRCA1* mutation; NC, patients with breast cancer but without the *BRCA1* mutation; PZ, generally healthy patients with the *BRCA1* mutation; PC, patients with both breast cancer and the *BRCA1* mutation.

Changes in TOS did not differ significantly between the groups, but TOS tended to be higher in the PC group (Figure 2).

**Figure 3:**
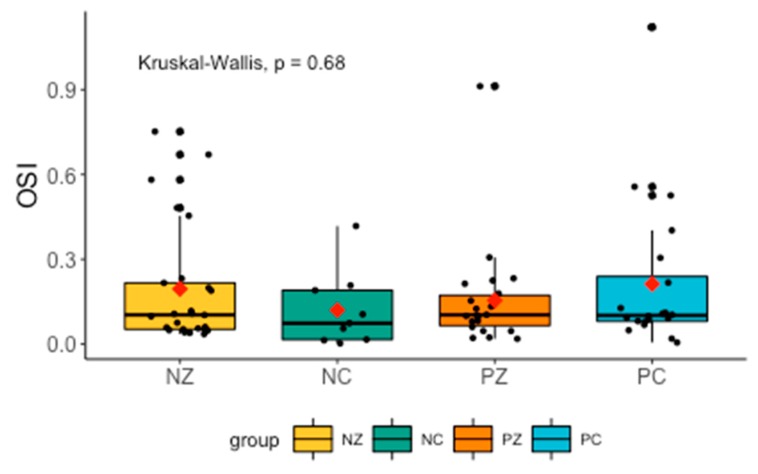
Levels of OSI in the saliva of the examined women. OSI, oxidative stress index; NZ, generally healthy patients without the *BRCA1* mutation; NC, patients with breast cancer but without the *BRCA1* mutation; PZ, generally healthy patients with the *BRCA1* mutation; PC, patients with both breast cancer and the *BRCA1* mutation.

Levels of OSI did not differ significantly between the groups (Figure 3).

**Figure 4:**
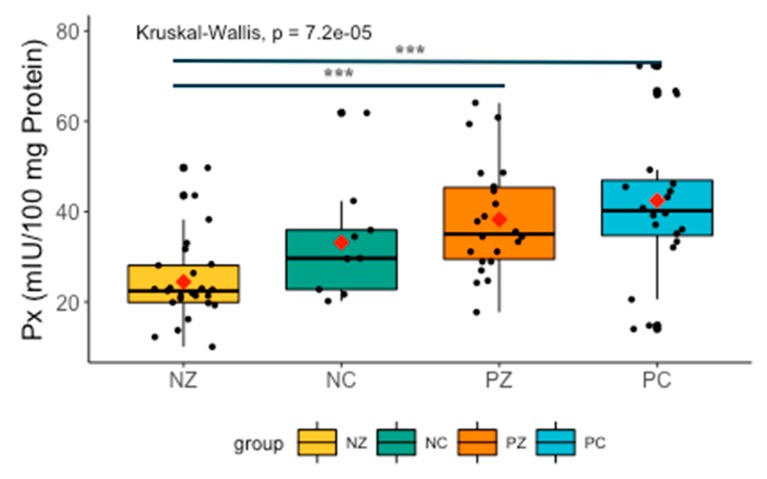
Activity of Px in the examined groups. Px, salivary peroxidase; NZ, generally healthy patients without the *BRCA1* mutation; NC, patients with breast cancer but without the *BRCA1* mutation; PZ, generally healthy patients with the *BRCA1* mutation; PC, patients with both breast cancer and the *BRCA1* mutation. *** *p* < 0.001.

Analysis revealed significant differences between all the examined groups. Px was higher in patients with both breast cancer and the *BRCA1* mutation compared to NZ (H = −4.246126, *p* = 0.0001), and slightly lower in PZ compared to NZ (H = −3.658388, *p* = 0.0004) (Figure 4).

**Figure 5:**
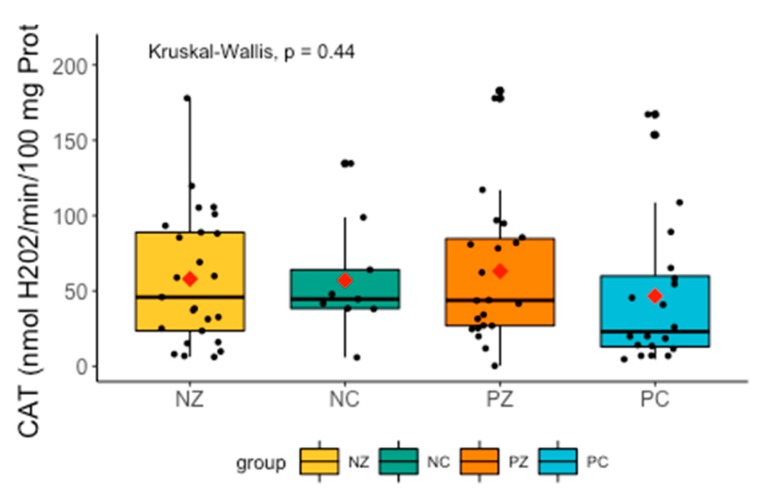
Activity of CAT in the examined groups. CAT, catalase activity; NZ, generally healthy patients without the *BRCA1* mutation; NC, patients with breast cancer but without the *BRCA1* mutation; PZ, generally healthy patients with the *BRCA1* mutation; PC, patients with both breast cancer and the *BRCA1* mutation.

There were no significant differences in CAT between the examined groups (Figure 5).

**Figure 6:**
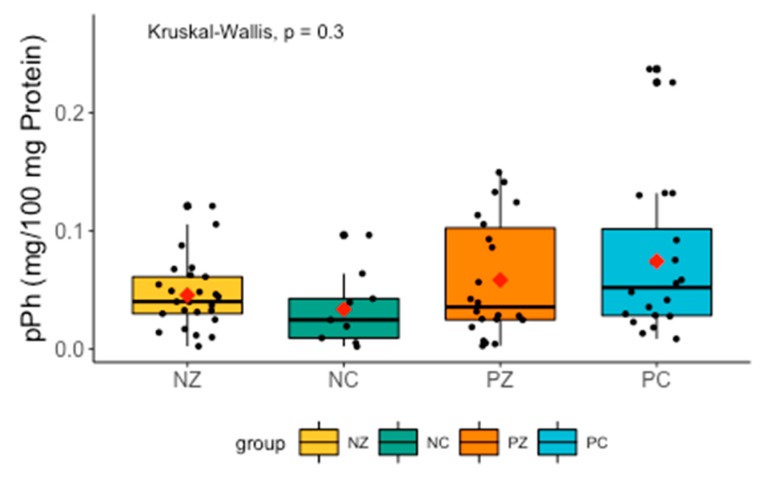
Levels of pPh in the examined groups. pPh, total phenolic content; NZ, generally healthy patients without the *BRCA1* mutation; NC, patients with breast cancer but without the *BRCA1* mutation; PZ, generally healthy patients with the *BRCA1* mutation; PC, patients with both breast cancer and the *BRCA1* mutation.

There were no significant differences in the levels of pPh between the examined groups. The level of pPh tended to be lower in women with breast cancer and without the *BRCA1* mutation (Figure 6).

**Figure 7:**
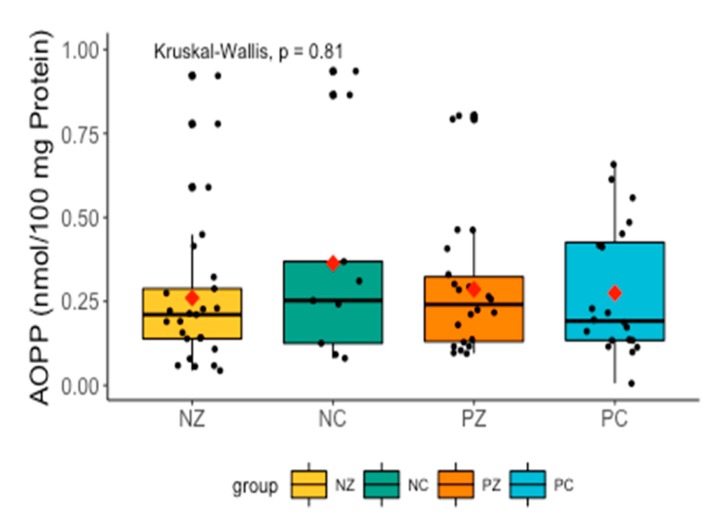
Levels of AOPP in the saliva of the examined women. AOPP, advanced oxidation protein products; NZ, generally healthy patients without the *BRCA1* mutation; NC, patients with breast cancer but without the *BRCA1* mutation; PZ, generally healthy patients with the *BRCA1* mutation; PC, patients with both breast cancer and the *BRCA1* mutation.

Levels of AOPP did not differ significantly between the examined groups (Figure 7).

The Kruskal–Wallis test revealed significant differences in the levels of AGE between the compared groups (H = 18.8335, *p* = 0.0000). Further analysis with the post-hoc Dunn’s test showed significant differences between NZ and PC (H = −4.2807856, *p* = 0.0001), and between NZ and PZ (H = −2.572300, *p* = 0.0152).

**Figure 8:**
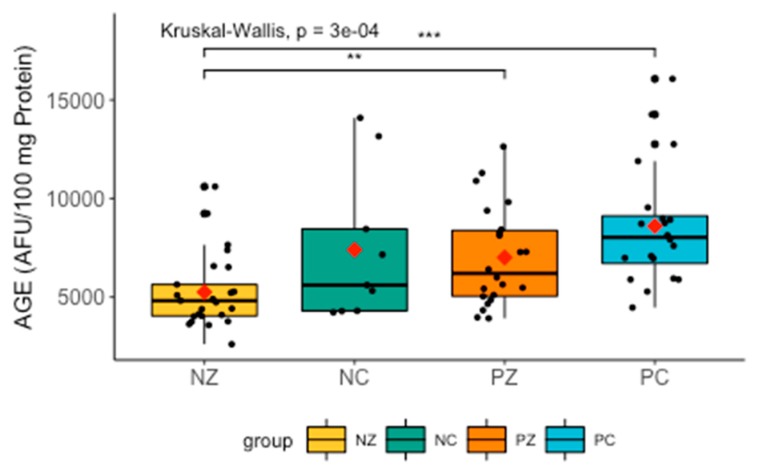
Levels of AGE in the examined groups. AGE, advanced glycation end-products; NZ, generally healthy patients without the *BRCA1* mutation; NC, patients with breast cancer but without the *BRCA1* mutation; PZ, generally healthy patients with the *BRCA1* mutation; PC, patients with both breast cancer and the *BRCA1* mutation. ** *p* < 0.01; *** *p* < 0.001.

There was a marked increase in AGE in patients with the *BRCA1* mutation and suffering from breast cancer. Levels of AGE were slightly increased in women with breast cancer without the *BRCA1* mutation, but the difference was insignificant (Figure 8).

**Figure 9:**
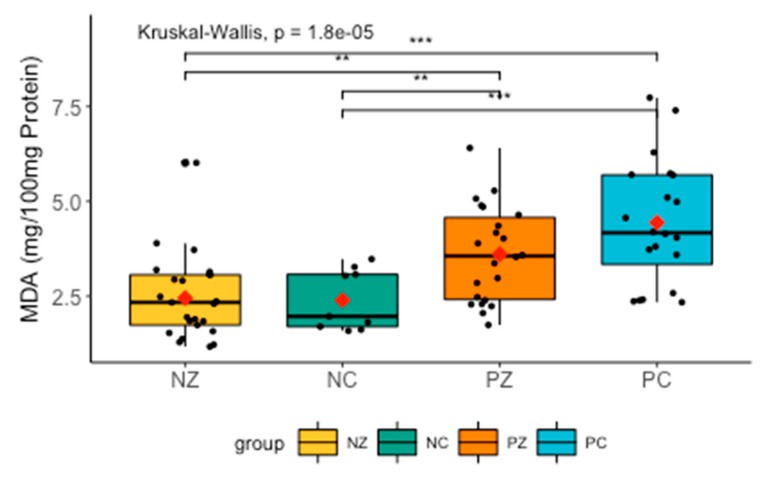
Levels of MDA in the saliva of the examined women. MDA, concentration of malondialdehyde; NZ, generally healthy patients without the *BRCA1* mutation; NC, patients with breast cancer but without the *BRCA1* mutation; PZ, generally healthy patients with the *BRCA1* mutation; PC, patients with both breast cancer and the *BRCA1* mutation. ** *p* < 0.01; *** *p* < 0.001.

There were significant differences in the levels of MDA between all groups. The post-hoc Dunn’s test revealed significantly higher levels of MDA between groups NC and PC (H = −3.365716, *p* = 0.001), NC and PZ (H = −2.285397, *p* = 0.0167), NZ and PC (H = −4.410597, *p* = 0.0000), as well as NZ and PZ (H = 2.998435, *p* = 0.0027) (Figure 9).

**Figure 10:**
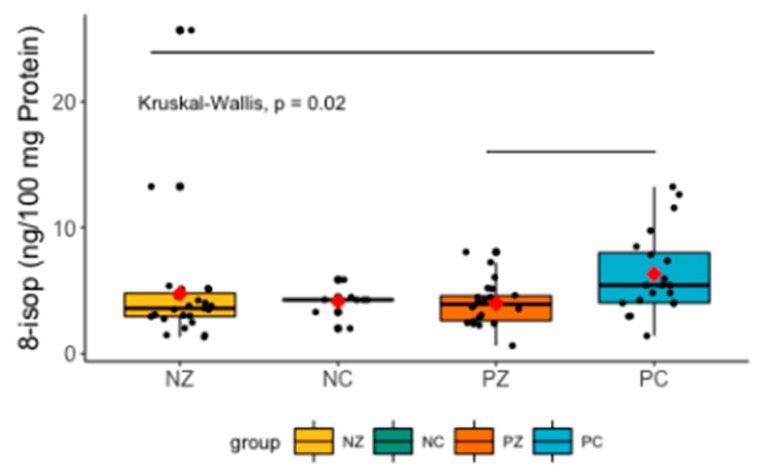
Levels of 8-isop in the saliva of the examined women. 8-isop, concentration of 8-isoprostane; NZ, generally healthy patients without the *BRCA1* mutation; NC, patients with breast cancer but without the *BRCA1* mutation; PZ, generally healthy patients with the *BRCA1* mutation; PC, patients with both breast cancer and the *BRCA1* mutation.

The Kruskal–Wallis test revealed significant differences between the compared groups (H = 9.7921, *p* = 0.02). The post-hoc Dunn’s test indicated significant differences in 8-isop levels between NZ and PC (H = 2.88315, *p* = 0.0118), and PC and PZ (H = 2.575711, *p* = 0.0150) (Figure 10).

**Figure 11:**
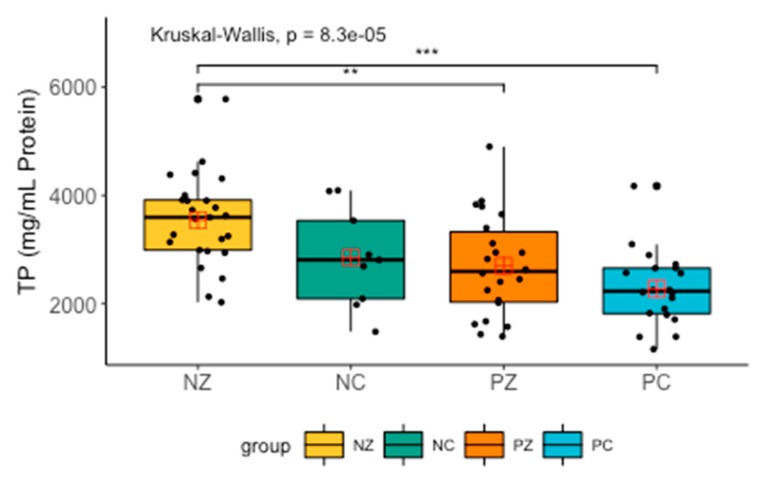
Levels of total protein in the saliva of the examined women. TP, total protein; NZ, generally healthy patients without the *BRCA1* mutation; NC, patients with breast cancer but without the *BRCA1* mutation; PZ, generally healthy patients with the *BRCA1* mutation; PC, patients with both breast cancer and the *BRCA1* mutation. ** *p* < 0.01; *** *p* < 0.001.

The Kruskal–Wallis test revealed significant differences in the levels of total protein between the compared groups (H = 21.496, *p* = 0.0000). Further analysis with the post-hoc Dunn’s test showed significantly lower levels of TP in the PZ and PC groups (H = 4.484560, *p* = 0.0000) compared to NZ and NC (H = 3.091942, *p* = 0.0030) (Figure 11).

### Correlations

**Figure 12:**
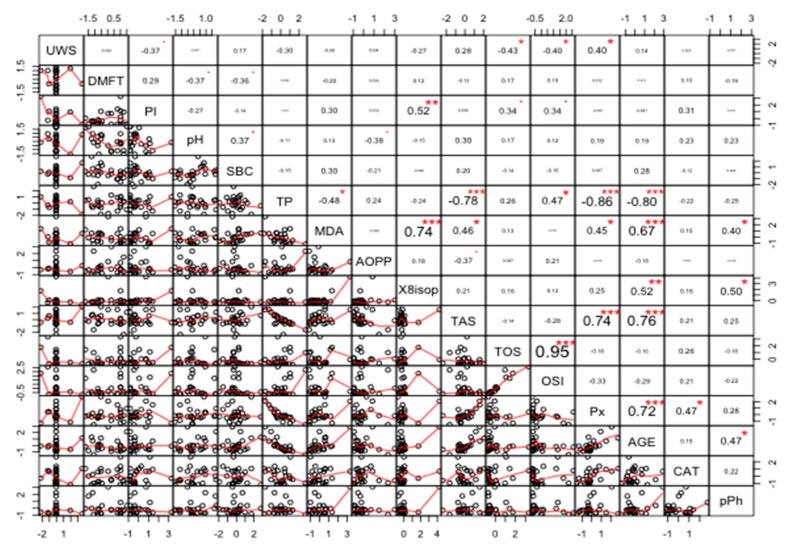
Coefficients of correlation between parameters measured in the saliva of healthy women without the *BRCA1* mutation (NZ). NZ, generally healthy patients without the *BRCA1* mutation; AOPP, advanced oxidation protein products; TP, total protein; OSI, oxidative stress index; TOS, total oxidant status; TAS, total antioxidant status; CAT, catalase activity; pPh, total phenolic content; Pl.I score, Plaque Index; DMFT, decay, missing, filled teeth; UWS, unstimulated whole saliva; SBC, salivary buffer capacity; 8-isop, concentration of 8-isoprostane; MDA, concentration of malondialdehyde; AGE, advanced glycation end-products; SBC, buffer capacity of saliva; Px, activity of salivary peroxidase. * *p* < 0.05; ** *p* < 0.01; *** *p* < 0.001.

The strongest positive correlation was found between TOS and OSI (r = 0.95, *p* < 0.00001). The strongest negative correlation was found between TP and Px (r = −0.86, *p* < 0.00001) (Figure 12).

**Figure 13:**
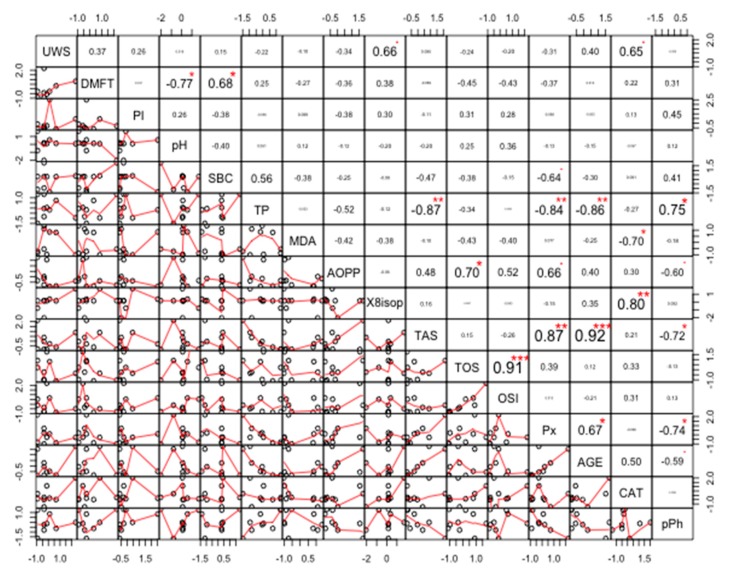
Coefficients of correlation between parameters analyzed in the saliva of women with breast cancer and without the *BRCA1* mutation (NC). NC, patients with breast cancer but without the *BRCA1* mutation; AOPP, advanced oxidation protein products; TP, total protein; OSI, oxidative stress index; TOS, total oxidant status; TAS, total antioxidant status; CAT, catalase activity; pPh, total phenolic content; Pl.I score, Plaque Index; DMFT, decay, missing, filled teeth; UWS, unstimulated whole saliva; SBC, salivary buffer capacity; 8-isop, concentration of 8-isoprostane; MDA, concentration of malondialdehyde; AGE, advanced glycation end-products; SBC, buffer capacity of saliva; Px, activity of salivary peroxidase. * *p* < 0.05; ** *p* < 0.01; *** *p* < 0.001.

The strongest positive correlations were found between TAS and AGE (r = 0.92, *p* < 0.0005), and TOS and OSI (r = 0.91, *p* < 0.0008). The strongest negative correlations were found between TP and TAS (r = −0.87, *p* < 0.0021), and between TP and AGE (r = −0.86, *p* < 0.0026) (Figure 13).

**Figure 14:**
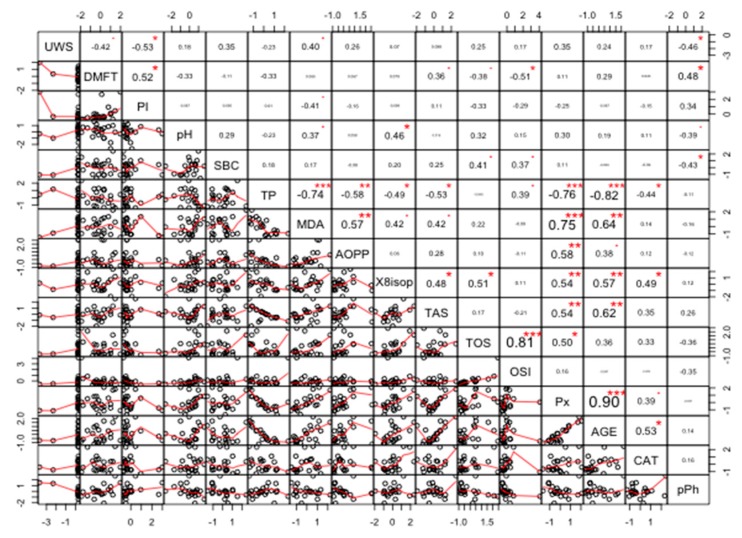
Coefficients of correlation between parameters measured in the saliva of women with the *BRCA1* mutation (PZ). PZ, generally healthy patients with the *BRCA1* mutation; AOPP, advanced oxidation protein products; TP, total protein; OSI, oxidative stress index; TOS, total oxidant status; TAS, total antioxidant status; CAT, catalase activity; pPh, total phenolic content; Pl.I score, Plaque Index; DMFT, decay, missing, filled teeth; UWS, unstimulated whole saliva; SBC, salivary buffer capacity; 8-isop, concentration of 8-isoprostane; MDA, concentration of malondialdehyde; AGE, advanced glycation end-products; SBC, buffer capacity of saliva; Px, activity of salivary peroxidase. * *p* < 0.05; ** *p* < 0.01; *** *p* < 0.001.

The strongest positive correlations were found between Px and AGE (r = 0.90, *p* < 0.00001), and TOS and OSI (r = 0.81, *p* < 0.00001). The strongest negative correlations were found between TP and AGE (r = −0.82, *p* < 0.00001), and between TP and Px (r = −0.76, *p* < 0.00001) (Figure 14).

**Figure 15:**
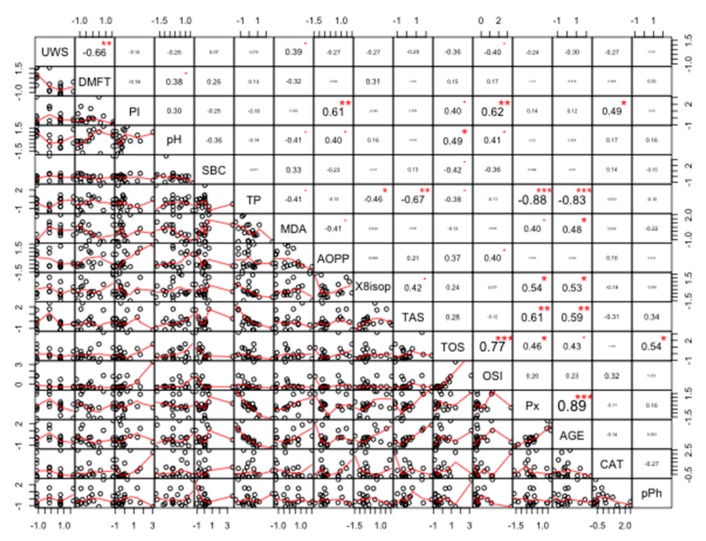
Coefficients of correlation between parameters analyzed in the saliva of women with both breast cancer and the *BRCA1* mutation (PC). PC, patients with both breast cancer and the *BRCA1* mutation. AOPP, advanced oxidation protein products; TP, total protein; OSI, oxidative stress index; TOS, total oxidant status; TAS, total antioxidant status; CAT, catalase activity; pPh, total phenolic content; Pl.I score, Plaque Index; DMFT, decay, missing, filled teeth; UWS, unstimulated whole saliva; SBC, salivary buffer capacity; 8-isop, concentration of 8-isoprostane; MDA, concentration of malondialdehyde; AGE, advanced glycation end-products; SBC, buffer capacity of saliva; Px, activity of salivary peroxidase. * *p* < 0.05; ** *p* < 0.01, *** *p* < 0.001.

Data shown in Figure 15 indicate a significant correlation between most parameters (at significance level alpha = 0.05). The strongest positive correlations were found between Px and AGE (r = 0.89, *p* < 0.00001), and TOS and OSI (r = 0.77, *p* < 0.00001). The strongest negative correlations were found between TP and AGE (r = −0.83, *p* < 0.00001), and TP and Px (r = −0.88, *p* < 0.00001) (Figure 15). The exact r and *p* values are given for all correlations in the Supplementary Material (Tables S1–S4).

**Figure 16:**
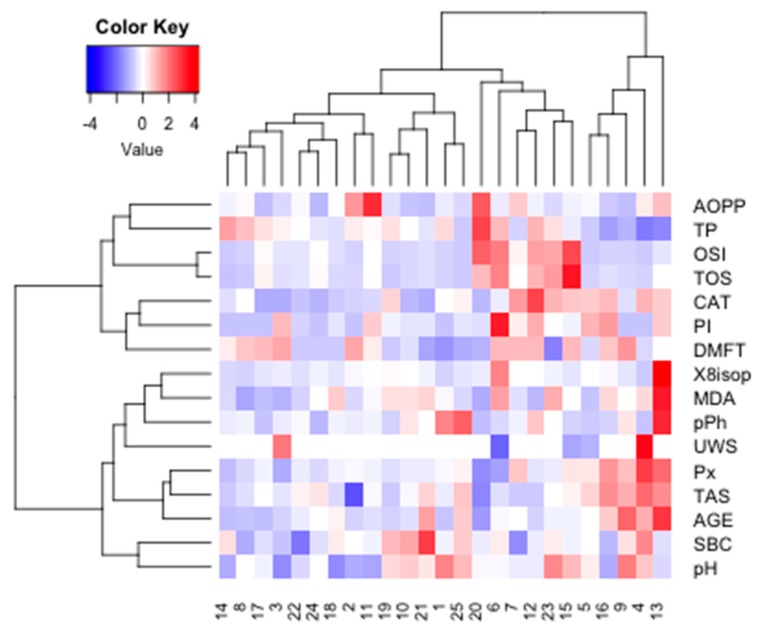
Cluster analysis for group NZ. NZ, generally healthy patients without the *BRCA1* mutation. AOPP, advanced oxidation protein products; TP, total protein; OSI, oxidative stress index; TOS, total oxidant status; TAS, total antioxidant status; CAT, catalase activity; pPh, total phenolic content; Pl.I score, Plaque Index; DMFT, decay, missing, filled teeth; UWS, unstimulated whole saliva; SBC, salivary buffer capacity; 8-isop, concentration of 8-isoprostane; MDA, concentration of malondialdehyde; AGE, advanced glycation end-products; SBC, buffer capacity of saliva; Px, activity of salivary peroxidase.

Cluster analysis was performed in the four examined groups of women to identify similarities between parameters measured in saliva (Figure 16).

The optimal number of clusters identified using the silhouette method allowed us to distinguish two main clusters for NZ data (*k* = 2). The similarity index was 0.15 for cluster 1 (AOPP, TP, OSI, TOS, CAT) and 0.20 for cluster 2 (8-isop, MDA, TPC, pPh, Px, TAS, AGE). The strongest similarity was found within cluster 2.

**Figure 17:**
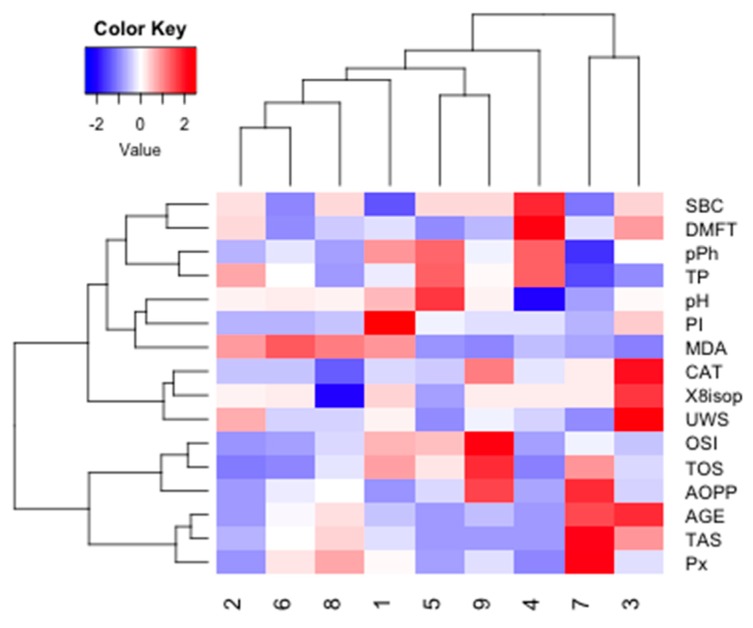
Cluster analysis for group NC. NC, patients with breast cancer but without the *BRCA1* mutation. AOPP, advanced oxidation protein products; TP, total protein; OSI, oxidative stress index; TOS, total oxidant status; TAS, total antioxidant status; CAT, catalase activity; pPh, total phenolic content; Pl.I score, Plaque Index; DMFT, decay, missing, filled teeth; UWS, unstimulated whole saliva; SBC, salivary buffer capacity; 8-isop, concentration of 8-isoprostane; MDA, concentration of malondialdehyde; AGE, advanced glycation end-products; SBC, buffer capacity of saliva; Px, activity of salivary peroxidase.

The optimal number of clusters identified using the silhouette method allowed us to distinguish four main clusters for NC data (*k* = 2). The similarity index was 0.09 for cluster 1 (SBC, TPC, TP, Ph, PI, MDA, CAT, ISOP, UWS) and 0.24 for cluster 2 (OSI, TOS, AOPP, AGE, TAS, Px). The strongest similarity was found within cluster 2 (Figure 17).

**Figure 18:**
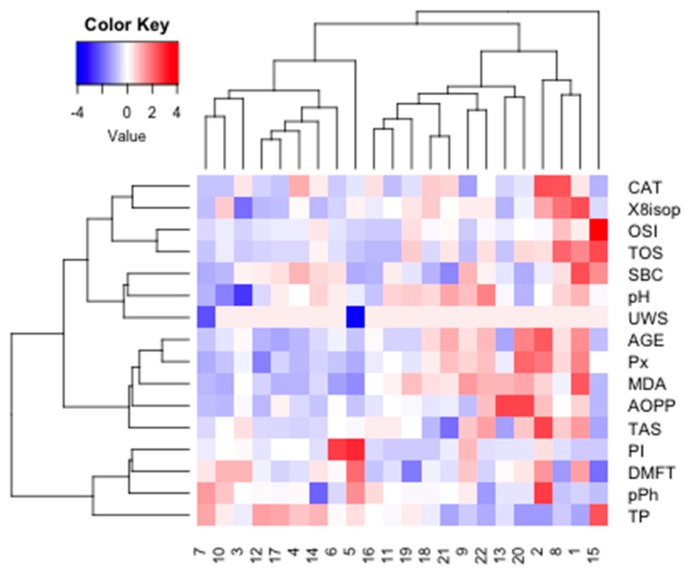
Cluster analysis for group PZ. PZ, generally healthy patients with the *BRCA1* mutation; AOPP, advanced oxidation protein products; TP, total protein; OSI, oxidative stress index; TOS, total oxidant status; TAS, total antioxidant status; CAT, catalase activity; pPh, total phenolic content; Pl.I score, Plaque Index; DMFT, decay, missing, filled teeth; UWS, unstimulated whole saliva; SBC, salivary buffer capacity; 8-isop, concentration of 8-isoprostane; MDA, concentration of malondialdehyde; AGE, advanced glycation end-products; SBC, buffer capacity of saliva; Px, activity of salivary peroxidase.

The optimal number of clusters identified using the silhouette method allowed us to distinguish four main clusters for PZ data (*k* = 3). The similarity index was 0.18 for cluster 1 (CAT, ISOP, OSI, TOS, SBC, pH, UWS), 0.18 for cluster 2 (AGE, Px, MDA, AOPP, TAS), and 0.28 for cluster 3 (PI, DMFT, pPh, TP). The strongest similarity was found within cluster 3 (Figure 18).

**Figure 19:**
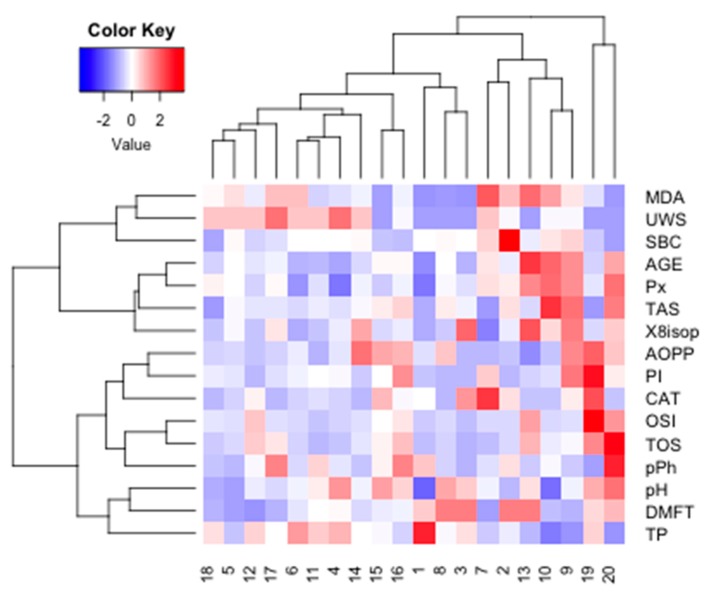
Cluster analysis for group PC. PC, patients with breast cancer and with the *BRCA1* mutation; AOPP, advanced oxidation protein products; TP, total protein; OSI, oxidative stress index; TOS, total oxidant status; TAS, total antioxidant status; CAT, catalase activity; pPh, total phenolic content; Pl.I score, Plaque Index; DMFT, decay, missing, filled teeth; UWS, unstimulated whole saliva; SBC, salivary buffer capacity; 8-isop, concentration of 8-isoprostane; MDA, concentration of malondialdehyde; AGE, advanced glycation end-products; SBC, buffer capacity of saliva; Px, activity of salivary peroxidase.

The optimal number of clusters identified using the silhouette method allowed us to distinguish four main clusters for PZ data (*k* = 4). The similarity index was 0.02 for cluster 1 (MDA, UWS, SBC), 0.22 for cluster 2 (AGE, Px, TAS, ISOP), 0.17 for cluster 3 (AOPP, PI, CAT, OSI, TOS, pPh), and 0.07 for cluster 4 (pH, DMFT, TP). The strongest similarity was found within cluster 2 (Figure 19).

**Figure 20:**
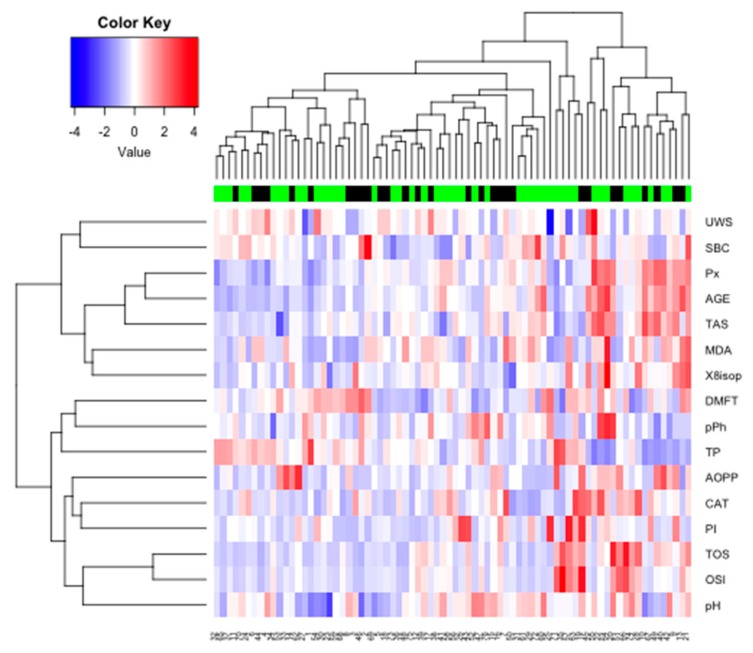
Heat map for all examined subjects. AOPP, advanced oxidation protein products; TP, total protein; OSI, oxidative stress index; TOS, total oxidant status; TAS, total antioxidant status; CAT, catalase activity; pPh, total phenolic content; Pl.I score, Plaque Index; DMFT, decay, missing, filled teeth; UWS, unstimulated whole saliva; SBC, salivary buffer capacity; 8-isop, concentration of 8-isoprostane; MDA, concentration of malondialdehyde; AGE, advanced glycation end-products; SBC, buffer capacity of saliva; Px, activity of salivary peroxidase.

The optimal number of clusters identified using the silhouette method allowed us to distinguish two main clusters (*k* = 2) for the whole group. The similarity index was 0.14 for cluster 1 (UWS, SBC, Px, AGE, TAS, MDA, ISOP) and 0.29 for cluster 2 (pPh, TP, AOPP, CAT, TOS, OSI). The strongest similarity was found within cluster 2 (Figure 20).

## 4. Discussion

This is the first study to evaluate salivary gland function, antioxidant defence and oxidative damage in unstimulated saliva of patients with breast cancer. We demonstrated that saliva from breast cancer patients with the *BRCA1* mutation is characterized by increased antioxidant potential and a higher degree of oxidative damage to proteins and lipids. In addition, our findings suggest that the *BRCA1* mutation can cause a predisposition to early salivary gland dysfunction, both in patients with breast cancer and in healthy individuals, leading to a decrease in the concentration of salivary proteins.

Changes in the quantitative and qualitative composition of saliva [10], as well as increased incidence of periodontal disease [9,10], have been reported in patients with breast cancer. It is very likely that oxidative stress plays an important role in the pathogenesis of these medical conditions. Indeed, redox imbalance has been regarded as one of the key factors responsible for the development of periodontitis and dysfunction in salivary glands in systemic diseases and cancer [18,22,28,29]. Oxidative stress can be detected by assessing the synthesis rate of reactive oxygen species, the concentration/activity of enzymatic and non-enzymatic antioxidants, and the level of oxidative products of protein and lipid modification. Although disorders of antioxidant defence mechanisms (↓reduced glutathione, ↑superoxide dismutase, ↓glutathione peroxidase) and intensity of oxidative cell damage (↑MDA, ↑protein carbonyl groups, ↑8-OHdG) were observed in the blood serum of patients with *BRCA1*-dependent breast cancer [6,7,8,30], no data are available on the capacity of antioxidant systems and biomarkers of oxidative stress in the saliva of patients with breast cancer and their relationship with salivary gland function.

The present study is the first to assess the activity of salivary antioxidant enzymes (CAT, Px), concentration of polyphenols, and total antioxidant status (TAS). Of all these parameters, TAS has particular clinical value since it allows us to determine the combined capacity of all antioxidant systems in preventing oxidative damage. Indeed, the total antioxidant effect is not a simple sum of all the antioxidants individually, but takes into account the interactions between antioxidants [31,32]. Our study demonstrated that the TAS in the saliva of breast cancer patients with the *BRCA1* mutation was significantly higher compared to controls. This suggests an adaptive response of the body to increased ROS production in *BRCA1*^+^ patients with breast cancer. Considering the fact that uric acid (UA) has the strongest contribution to TAS [31,33], an increase in TAS may suggest an increased secretion of this most important antioxidant in the oral cavity. UA determines about 70–80% of the antioxidant capacity of plasma and saliva [33,34]. This compound is produced in the salivary glands, although its largest quantities pass into the saliva from blood plasma as a by-product of purine metabolism [33,34]. It is well known that an increased plasma concentration of UA is detected in patients with myeloproliferative and lymphoproliferative disorders, cancer, and during chemotherapy and radiotherapy (tumor lysis syndrome) [35]. Considering that increased plasma UA levels have been found in women with breast cancer [35,36], this compound may pass from plasma to saliva, and thus contribute to increased salivary TAS. However, we found no changes in TAS levels in *BRCA1*– patients with breast cancer compared to controls. This indicates that breast cancer and the *BRCA1* mutation (and not cancer alone) are associated with the induction of antioxidant mechanisms.

In our study, CAT activity did not differ between the groups, while the activity of salivary Px was significantly higher in the *BRCA1*^+^ breast cancer patients as compared to controls. Salivary peroxidase, because of its functions, is regarded as the most important salivary enzyme. This compound has a dual role: it is responsible for the breakdown of cytotoxic hydrogen peroxide, and has bactericidal activity against *Streptococcus mutans, Escherichia coli, Salmonella typhimurium* and various *Lactobacillus* and *Actinomyces* species [31,33]. It is worth emphasizing that salivary peroxidase is the only antioxidant synthesized exclusively in the salivary glands [31,33]. Thus, the activity of salivary peroxidase reflects the efficiency of the salivary glands in preventing oxidative stress. Analysis of the data obtained in our study suggests that the increase in the activity of Px in the saliva of *BRCA1*^+^ patients with breast cancer indicates an intensified enzymatic antioxidant defence, protecting against oxidative damage to salivary glands and the entire oral cavity. This hypothesis is supported by the positive correlation between the activity of Px and the level of AGE in these patients (r = 0.89, *p* < 0.00001 in the PC group).

Despite intensified mechanisms of antioxidant defence (↑TAS, ↑Px), oxidative damage to salivary proteins (↑AGE) and lipids (↑MDA) is observed in patients with both breast cancer and the *BRCA1* mutation. It is well known that MDA is a compound with high toxic and mutagenic potential, and may contribute to the onset and progression of cancer [37]. MDA disturbs the structure of phospholipids in cell membranes and reacts with the nitrogenous bases present in DNA and RNA by creating adducts. Complexes formed in this reaction may cause genomic instability, replication errors and abnormal cell signal transduction [27,37]. Moreover, MDA can interact with proteins and peptides, and can disrupt many metabolic processes [27,37]. Indeed, the accumulation of oxidized proteins in the oral cavity/salivary glands (e.g., AGE) may stimulate their aggregation and increase the synthesis of pro-inflammatory cytokines (IL-1, IL-6). It has been shown that AGE, through interaction with a specific receptor (RAGE), not only enhance inflammation, but are also responsible for the overproduction of ROS through the activation of nicotinamide adenine dinucleotide phosphate (NADPH) oxidase (NOX)—the main source of ROS in the cell [19,38,39]. In addition, proteins modified by oxidation lose their biological functions and are degraded or accumulated [19,39]. However, we found no significant differences in the levels of other oxidative stress biomarkers (AOPP, 8-isop, TOS and OSI), which suggests that the body may, to some degree, defend itself against damage caused by reactive oxygen species. We found no changes in the concentrations of oxidative modification products in breast cancer patients without the *BRCA1* mutation, which corroborates our earlier observations regarding the relationship between cancer, the *BRCA1* mutation, and disorders of redox balance.

How does the *BRCA1* mutation disturb the salivary redox profile in breast cancer patients? The *BRCA1* gene is expressed in many tissues and organs, such as the breast, ovaries, testes, lymphocytes, brain and sigmoid colon. It was also detected in salivary glands and other regions of the oral cavity [40]. The BRCA1 protein plays an important role in maintaining genome integrity: it is involved in the control of transcription, the repair of DNA double-strand breaks, and cell cycle regulation [2]. Importantly, in response to DNA damage, BRCA1 undergoes phosphorylation dependent on the ATM (Ataxia Telangiectasia) protein. In fact, ATM is necessary for the activation of the response to oxidative DNA damage, but the phosphorylation of ATM activates a number of proteins associated with apoptosis and cell cycle arrest, such as p-53 or c-Abl kinase [3,4]. However, ATM also reduces ROS production by being responsible for the regeneration of thioredoxin, the key intracellular antioxidant [41]. It is well known that mutations of the *ATM* gene cause ataxia telangiectasia, manifested by genomic instability, disorders of cell proliferation, and increased sensitivity to DNA damage [42]. Thus, *ATM* mutations cause similar alterations at the cellular level to *BRCA1* mutations. Furthermore, an increased predisposition to many cancers, including a 1.3–12.7-fold higher risk of breast cancer, was reported in patients with ataxia telangiectasia [43]. A key role in the aetiology of ataxia telangiectasia (both at the molecular and systemic levels) is attributed to oxidative stress [41]. Supposedly, disturbances of redox balance play the same role in patients with breast cancer. Although many in vitro studies have demonstrated an overproduction of ROS and increased free radical damage caused by the *BRCA1* mutation [3,4], the mechanisms responsible for the induction of oxidative stress in patients with breast cancer have not been explained in detail. Presumably, by binding to other proteins (including ATM), BRCA1 not only allows transcription/cell cycle regulation, but is also involved in maintaining the redox status of the cell [3,4]. However, at the moment it is impossible to determine whether the *BRCA1* mutation in patients with breast cancer is the cause or effect of the oxidative stress. Therefore, we indicate the need for further studies to explain the role of redox balance disturbances in the pathogenesis of breast cancer, because the abnormalities in redox balance observed in our patients are caused not only by cancer but also by the *BRCA1* mutation.

In our study, we assessed oxidative stress biomarkers and also evaluated the secretory function of the salivary glands by measuring the salivary flow and total protein concentration. Although we did not demonstrate changes in unstimulated salivary secretion between different groups of patients, we found a significant decrease in the level of salivary proteins in all patients with the *BRCA1* mutation (both healthy subjects and those with breast cancer) compared to healthy women without the mutation. Interestingly, no changes in total protein levels were found in breast cancer patients with the *BRCA1* mutation. It is well known that a decrease in salivary protein concentration is a sensitive marker of impaired secretory function of the salivary glands [44]. Although our research does not explain the cause of the observed alterations, it can be assumed that the early hypofunction of the salivary glands in subjects with the *BRCA1* mutation may be induced by oxidative stress. This is indicated by the negative correlation between total protein content and the content of AGE (r = −0.83, *p* < 0.00001), MDA (r = −0.41, *p* = 0.0454), and TAS (r = −0.67, *p* = 0.0013) in the unstimulated saliva of *BRCA1*^+^ patients with breast cancer.

Researchers increasingly often emphasize the role of saliva as a non-invasive, alternative diagnostic material to blood and other body fluids [45]. Indeed, saliva sampling is a low-cost procedure, does not require the involvement of specialist medical staff, and the concentration of most of the substances present in saliva correlate with their content in blood plasma [18]. The diagnostic value of salivary redox biomarkers has been demonstrated in the diagnosis of metabolic diseases (obesity, insulin resistance [15,17]), inflammatory diseases (chronic kidney disease, gingivitis [18,29]) and neurodegenerative diseases (dementia, Alzheimer’s disease [19,20]). Our study also indicates the potential suitability of the salivary parameters of oxidative stress in the differential diagnosis of breast cancer patients with/without the *BRCA1* mutation. Using cluster analysis, we identified parameters that may have particular clinical significance and are characterized by the highest degree of similarity within the studied groups. Of all these parameters, salivary peroxidase, AGE, TAS and MDA deserve special consideration. Nevertheless, the diagnostic value of redox biomarkers should be assessed in a larger population of patients. The analysis of saliva–blood correlation is also indicated.

Our study has some limitations. Firstly, we evaluated only selected salivary antioxidants and oxidative damage products, which means that we cannot fully conclude on the salivary redox balance in patients with breast cancer. Secondly, we only analyzed redox biomarkers in unstimulated saliva and did not conduct a complete periodontological examination. However, the unquestionable advantage of this study was the careful selection (in terms of inclusion and exclusion criteria) of the experimental and control groups, and the fact that it is the first study to evaluate the antioxidant barrier and oxidative damage in the saliva of patients with *BRCA1*-mediated and *BRCA1*-unmediated breast cancer. Further research is needed to clarify the molecular basis of redox imbalance in subjects with breast cancer. We believe that it is advisable to evaluate the expression of proteins involved in protection against oxidative damage such as ATM and ATR (Ataxia telangiectasia Rad 3-related), as well as thioredoxin-1 and glutathione. The next step would be to assess the saliva–blood correlations of the assessed redox biomarkers. Consequently, our research is a starting point for future basic and clinical research.

## 5. Conclusions

The presence of the *BRCA1* mutation in breast cancer patients was associated with a stronger antioxidant barrier in saliva, and such alterations were not observed in cancer patients without the *BRCA1* mutation. Oxidative damage to salivary proteins and lipids was increased in patients with breast cancer. The *BRCA1* mutation, in both healthy subjects and breast cancer patients, may predispose them to impaired secretory function of the salivary glands.

## Figures and Tables

**Table 1:** Characteristics of the analyzed groups.

	NZ (*n* = 25)	NC (*n* = 9)	PZ (*n* = 22)	PC (*n* = 20)	Kruskal–Wallis (*P*)
Mean age	39.73	43.33	43.40	48.70	0.07
Median age	41.00	42.00	41.00	48.00	0.07
*Reproductive status*					
Reproductive	64.00%	55.58%	68.18%	45.00%	NA
Premenopausal	8.00%	11.11%	4.55%	10.00%	NA
Menopausal	28.00%	11.11%	27.27%	25.00%	NA
Postmenopausal	0.00%	22.2%	0.00%	20.00%	NA
Mean age at menarche	13.92	14.44	14.05	13.80	0.84
*Fertility rate*					
0 children	12.00%	11.11%	18.18%	25.00%	NA
1–2 children	56.00%	77.78%	54.55%	45.00%	NA
>2 children	32.00%	11.11%	27.27%	30.00%	NA
Mean number of children	1.84	2.22	1.95	1.85	0.98
*Cancer location*					
Left breast	no cancer	33.33%	no cancer	45.00%	NA
Right breast	no cancer	55.56%	no cancer	45.00%	NA
Both breasts	no cancer	11.11%	no cancer	10.00%	NA
*Cancer type*					
Ductal	0.0%	100.00%	0.0%	100.00%	NA
Lobular	0.0%	0%	0.0%	0.0%	NA

NZ, generally healthy patients without the *BRCA1* mutation; NC, patients with breast cancer but without the *BRCA1* mutation; PC, patients with both breast cancer and the *BRCA1* mutation; PZ, generally healthy patients with the *BRCA1* mutation; NA, not applicable.

**Table 2:** Secretory function of salivary glands and selected dental indices.

	NZ (*n* = 25)	NC (*n* = 9)	PZ (*n* = 22)	PC (*n* = 20)
pH	7.20 (7.62–6.61)	7.21 (7.72–6.80)	7.30 (7.83–6.84)	7.10 (7.80–5.98)
DMFT	5.0 (10.0–3.7)	4.7 (6.5–2.5)	5.5 (10.00–2.8)	4.2 (6.9–1.7)
Pl.I	0.25 (3.33–0.00)	0.30 (2.60–0.00)	0.43 (2.60–0.00)	0.49 (5.55–0.00)
SBC	6.57 (9.37–3.52)	7.30 (15.00–1.87)	6.44 (20.00–2.14)	6.75 (14.28–2.00)
UWS flow rate	0.27 (0.61–0.20)	0.33 (0.55–0.20)	0.27 (0.40–0.20)	0.33 (0.33–0.13)

DMFT, decay, missing, filled teeth; NZ, generally healthy patients without the *BRCA1* mutation; NC, patients with breast cancer but without the *BRCA1* mutation; UWS, unstimulated whole saliva; Pl.I score, Plaque Index; PC, patients with both breast cancer and the *BRCA1* mutation; PZ, generally healthy patients with the *BRCA1* mutation; SBC, salivary buffer capacity.

## Supplementary Materials

The following are available online at https://www.mdpi.com/2072-6694/11/10/1501/s1, Table S1: R and *p*-value of correlation between parameters analysed in the saliva of women with breast cancer and with the BRCA1 mutation (PC), Table S2: R and *p* value of correlation between parameters measured in the saliva of women with the BRCA1 mutation (PZ), Table S3: R and *p* value of correlation between parameters measured in the saliva of healthy women without the BRCA1 mutation (NZ), Table S4: R and *p* value of correlation between parameters analysed in the saliva of women with breast cancer and without the BRCA1 mutation (NC).

## References

[1] Winters S., Martin C., Murphy D., Shokar N.K. (2017). Breast cancer epidemiology, prevention, and screening. Progress in Molecular Biology and Translational Science.

[2] Huszno J., Kołosza Z., Grzybowska E. (2019). BRCA1 mutation in breast cancer patients: Analysis of prognostic factors and survival. Oncol. Lett..

[3] Marks J.R. (2013). Refining the role of BRCA1 in combating oxidative stress. Breast Cancer Res..

[4] Yi Y.W., Kang H.J., Bae I. (2014). BRCA1 and oxidative stress. Cancers.

[5] Valko M., Leibfritz D., Moncol J., Cronin M.T.D., Mazur M., Telser J. (2007). Free radicals and antioxidants in normal physiological functions and human disease. Int. J. Biochem. Cell Biol..

[6] Rajneesh C.P., Manimaran A., Sasikala K.R., Adaikappan P. (2008). Lipid peroxidation and antioxidant status in patients with breast cancer. Singap. Med. J..

[7] Ray G., Batra S., Shukla N.K., Deo S., Raina V., Ashok S., Husain S.A. (2000). Lipid peroxidation, free radical production and antioxidant status in breast cancer. Breast Cancer Res. Treat..

[8] Gönenç A., Erten D., Aslan S., Akinci M., Şimşek B., Torun M. (2006). Lipid peroxidation and antioxidant status in blood and tissue of malignant breast tumor and benign breast disease. Cell Biol. Int..

[9] Söder B., Yakob M., Meurman J.H., Andersson L.C., Klinge B., Söder P.Ö. (2011). Periodontal disease may associate with breast cancer. Breast Cancer Res. Treat..

[10] Emekli-Alurfan E., Demir G., Kasikci E., Tunali-Akbay T., Pisiriciler R., Caliskan E., Yarat A. (2008). Altered biochemical parameters in the saliva of patients with breast cancer. Tohoku J. Exp. Med..

[11] Gornitsky M., Velly A.M., Mohit S., Almajed M., Su H., Panasci L., Schipper H.M. (2016). Altered levels of salivary 8-oxo-7-hydrodeoxyguanosine in breast cancer. JDR Clin. Transl. Res..

[12] Hecht F., Pessoa C.F., Gentile L.B., Rosenthal D., Carvalho D.P., Fortunato R.S. (2016). The role of oxidative stress on breast cancer development and therapy. Tumor Biol..

[13] Gurer-Orhan H., Ince E., Konyar D., Saso L., Suzen S. (2017). The role of oxidative stress modulators in breast cancer. Curr. Med. Chem..

[14] Zalewska A., Ziembicka D., Żendzian-Piotrowska M., Maciejczyk M. (2019). The impact of high-fat diet on mitochondrial function, free radical production, and nitrosative stress in the salivary glands of wistar rats. Oxid. Med. Cell. Longev..

[15] Kołodziej U., Maciejczyk M., Miasko A., Matczuk J., Knas M., Zukowski P., Zendzian-Piotrowska M., Borys J., Zalewska A. (2017). Oxidative modification in the salivary glands of high fat-diet induced insulin resistant rats. Front. Physiol..

[16] Porto-Mascarenhas E.C., Assad D.X., Chardin H., Gozal D., De Luca Canto G., Acevedo A.C., Guerra E.N.S. (2017). Salivary biomarkers in the diagnosis of breast cancer: A review. Crit. Rev. Oncol. Hematol..

[17] Fejfer K., Buczko P., Niczyporuk M., Ładny J.R., Hady H.R., Knaś M., Waszkiel D., Klimiuk A., Zalewska A., Maciejczyk M. (2017). Oxidative modification of biomolecules in the nonstimulated and stimulated saliva of patients with morbid obesity treated with bariatric surgery. Biomed. Res. Int..

[18] Maciejczyk M., Szulimowska J., Skutnik A., Taranta-Janusz K., Wasilewska A., Wiśniewska N., Zalewska A. (2018). Salivary biomarkers of oxidative stress in children with chronic kidney disease. J. Clin. Med..

[19] Klimiuk A., Maciejczyk M., Choromańska M., Fejfer K., Waszkiewicz N., Zalewska A. (2019). Salivary redox biomarkers in different stages of dementia severity. J. Clin. Med..

[20] Choromańska M., Klimiuk A., Kostecka-Sochoń P., Wilczyńska K., Kwiatkowski M., Okuniewska N., Waszkiewicz N., Zalewska A., Maciejczyk M. (2017). Antioxidant defence, oxidative stress and oxidative damage in saliva, plasma and erythrocytes of dementia patients. Can salivary AGE be a marker of dementia?. Int. J. Mol. Sci..

[21] World Health Organization (2013). Oral Health Surveys: Basic Methods.

[22] Maciejczyk M., Skutnik-Radziszewska A., Zieniewska I., Matczuk J., Domel E., Waszkiel D., Żendzian-Piotrowska M., Szarmach I., Zalewska A. (2019). Antioxidant defense, oxidative modification, and salivary gland function in an early phase of cerulein pancreatitis. Oxid. Med. Cell. Longev..

[23] Mansson-Rahemtulla B., Baldone D.C., Pruitt K.M., Rahemtulla F. (1986). Specific assays for peroxidases in human saliva. Arch. Oral Biol..

[24] Aebi H. (1984). Catalase in vitro. Methods in Enzymology.

[25] Singleton V.L., Orthofer R., Lamuela-Raventós R.M. (1999). Analysis of Total Phenols and Other Oxidation Substrates and Antioxidants by Means of Folin-Ciocalteu Reagent. Methods in Enzymology.

[26] Kalousová M., Skrha J., Zima T. (2002). Advanced glycation end-products and advanced oxidation protein products in patients with diabetes mellitus. Physiol. Res..

[27] Buege J.A., Aust S.D. (1978). Microsomal lipid peroxidation. Methods Enzymol..

[28] Żukowski P., Maciejczyk M., Waszkiel D. (2018). Sources of free radicals and oxidative stress in the oral cavity. Arch. Oral Biol..

[29] Tothova L., Kamodyova N., Cervenka T., Celec P. (2015). Salivary markers of oxidative stress in oral diseases. Front. Cell. Infect. Microbiol..

[30] Pande D., Negi R., Karki K., Khanna S., Khanna R.S., Khanna H.D. (2012). Oxidative damage markers as possible discriminatory biomarkers in breast carcinoma. Transl. Res..

[31] Rice-Evans C., Miller N.J. (1994). [241 Total Antioxidant Status in Plasma and Body Fluids. Methods in Enzymology.

[32] Borys J., Maciejczyk M., Antonowicz B., Krętowski A., Waszkiel D., Bortnik P., Czarniecka-Bargłowska K., Kocisz M., Szulimowska J., Czajkowski M. (2018). Exposure to Ti4Al4V titanium alloy leads to redox abnormalities, oxidative stress, and oxidative damage in patients treated for mandible fractures. Oxid. Med. Cell. Longev..

[33] Knaś M., Maciejczyk M., Waszkiel D., Zalewska A. (2013). Oxidative stress and salivary antioxidants. Dent. Med. Probl..

[34] Nagler R.M., Klein I., Zarzhevsky N., Drigues N., Reznick A.Z. (2002). Characterization of the differentiated antioxidant profile of human saliva. Free Radic. Biol. Med..

[35] Fini M.A., Elias A., Johnson R.J., Wright R.M. (2012). Contribution of uric acid to cancer risk, recurrence, and mortality. Clin. Transl. Med..

[36] Yue C.F., Feng P.N., Yao Z.R., Yu X.G., Lin W.B., Qian Y.M., Guo Y.M., Li L.S., Liu M. (2017). High serum uric acid concentration predicts poor survival in patients with breast cancer. Clin. Chim. Acta.

[37] Ayala A., Muñoz M.F., Argüelles S. (2014). Lipid peroxidation: Production, metabolism, and signaling mechanisms of malondialdehyde and 4-hydroxy-2-nonenal. Oxid. Med. Cell. Longev..

[38] Maciejczyk M., Żebrowska E., Chabowski A. (2019). Insulin resistance and oxidative stress in the brain: What’s new?. Int. J. Mol. Sci..

[39] Wautier M.-P., Chappey O., Corda S., Stern D.M., Schmidt A.M., Wautier J.-L. (2001). Activation of NADPH oxidase by AGE links oxidant stress to altered gene expression via RAGE. Am. J. Physiol. Metab..

[40] Irani S., Bidari-Zerehpoush F. (2017). BRCA1/2 mutations in salivary pleomorphic adenoma and carcinoma-ex-pleomorphic adenoma. J. Int. Soc. Prev. Community Dent..

[41] Maciejczyk M., Mikoluc B., Pietrucha B., Heropolitanska—Pliszka E., Pac M., Motkowski R., Car H. (2017). Oxidative stress, mitochondrial abnormalities and antioxidant defense in Ataxia-telangiectasia, Bloom syndrome and Nijmegen breakage syndrome. Redox Biol..

[42] Lavin M.F., Scott S., Gueven N., Kozlov S., Peng C., Chen P. (2004). Functional consequences of sequence alterations in the ATM gene. DNA Repair.

[43] Angèle S., Hall J. (2000). The ATM gene and breast cancer: Is it really a risk factor?. Mutat. Res. Rev. Mutat. Res..

[44] Maciejczyk M., Kossakowska A., Szulimowska J., Klimiuk A., Car H., Ładny J.R., Chabowski A., Zalewska A. (2017). Lysosomal exoglycosidase profile and secretory function in the salivary glands of rats with streptozotocin-induced diabetes. J. Diabetes Res..

[45] Javaid M.A., Ahmed A.S., Durand R., Tran S.D. (2016). Saliva as a diagnostic tool for oral and systemic diseases. J. Oral Biol. Craniofacial Res..

