# Supplementary material for: Salivary Gland Function, Antioxidant Defence and Oxidative Damage in the Saliva of Patients with Breast Cancer: Does the BRCA1 Mutation Disturb the Salivary Redox Profile?
Source: Cancers (Basel). 2019 Oct 8;11(10):1501. doi: 10.3390/cancers11101501 (PMC6826655; doi:10.3390/cancers11101501)
Supplement: Supplementary file 1 [file cancers-11-01501-s001.pdf]

Supplementary Materials

Table S1. R and *p*-value of correlation between parameters analysed in the saliva of women with breast cancer and with the BRCA1 mutation (PC).

R

	UWS	DMFT	PI	pH	SBC	TP	MDA	AOPP	X8isop	TAS	TOS
**UWS**	1.00	−0.66	−0.14	−0.26	0.07	0.08	0.39	−0.27	−0.27	−0.20	−0.36
**DMFT**	−0.66	1.00	−0.18	0.38	0.26	0.13	−0.32	−0.04	0.31	−0.01	0.15
**PI**	−0.14	−0.18	1.00	0.30	−0.25	−0.18	0.04	0.61	−0.06	0.01	0.40
**pH**	−0.26	0.38	0.30	1.00	−0.36	−0.14	−0.41	0.40	0.16	0.00	0.49
**SBC**	0.07	0.26	−0.25	−0.36	1.00	−0.07	0.33	−0.23	−0.01	0.13	−0.42
**TP**	0.08	0.13	−0.18	−0.14	−0.07	1.00	−0.41	−0.13	−0.46	−0.67	−0.38
**MDA**	0.39	−0.32	0.04	−0.41	0.33	−0.41	1.00	−0.41	0.05	−0.04	−0.13
**AOPP**	−0.27	−0.04	0.61	0.40	−0.23	−0.13	−0.41	1.00	0.07	0.21	0.37
**X8isop**	−0.27	0.31	−0.06	0.16	−0.01	−0.46	0.05	0.07	1.00	0.42	0.24
**TAS**	−0.20	0.01	0.01	0.00	0.13	−0.67	−0.04	0.21	0.42	1.00	0.28
**TOS**	−0.36	0.15	0.40	0.49	−0.42	−0.38	−0.13	0.37	0.24	0.28	1.00
**OSI**	−0.40	0.17	0.62	0.41	−0.36	−0.11	−0.05	0.40	0.08	−0.12	0.77
**Px**	−0.24	0.00	0.14	−0.02	0.03	−0.88	0.40	0.01	0.54	0.61	0.46
**AGE**	−0.30	0.02	0.12	0.02	−0.02	−0.83	0.48	−0.03	0.53	0.59	0.43
**CAT**	−0.27	0.03	0.49	0.17	0.14	0.05	0.05	0.16	−0.19	−0.31	−0.01
**pPh**	−0.03	0.06	−0.02	0.16	−0.15	−0.10	−0.22	0.01	−0.09	0.34	0.54

	OSI	Px	AGE	CAT	pPh
**UWS**	−0.40	−0.24	−0.30	−0.27	−0.03
**DMFT**	0.17	0.00	0.02	0.03	0.06
**PI**	0.62	0.14	0.12	0.49	−0.02
**pH**	0.41	−0.02	0.02	0.17	0.16
**SBC**	−0.36	0.03	−0.02	0.14	−0.15
**TP**	−0.11	−0.88	−0.83	0.05	−0.10
**MDA**	−0.05	0.40	0.48	0.05	−0.22
**AOPP**	0.40	0.01	−0.03	0.16	0.01
**X8isop**	0.08	0.54	0.53	−0.19	−0.09
**TAS**	−0.12	0.61	0.59	−0.31	0.34
**TOS**	0.77	0.46	0.43	−0.01	0.54
**OSI**	1.00	0.20	0.23	0.32	0.03
**Px**	0.20	1.00	0.89	−0.11	0.16
**AGE**	0.23	0.89	1.00	−0.14	0.08
**CAT**	0.32	−0.11	−0.14	1.00	−0.27
**pPh**	0.03	0.16	0.08	−0.27	1.00

n = 20

P

	UWS	DMFT	PI	pH	SBC	TP	MDA	AOPP	X8isop
**UWS**		0.0015	0.5436	0.2704	0.7689	0.7501	0.0851	0.2464	0.2413
**DMFT**	0.0015		0.4426	0.0944	0.2596	0.5991	0.1627	0.8538	0.1892
**PI**	0.5436	0.4426		0.1910	0.2929	0.4382	0.8610	0.0046	0.8131
**pH**	0.2704	0.0944	0.1910		0.1186	0.5598	0.0763	0.0769	0.4985
**SBC**	0.7689	0.2596	0.2929	0.1186		0.7659	0.1548	0.3191	0.9570
**TP**	0.7501	0.5991	0.4382	0.5598	0.7659		0.0754	0.5987	0.0396
**MDA**	0.0851	0.1627	0.8610	0.0763	0.1548	0.0454		0.0754	0.8214

**AOPP**	0.2464	0.8538	0.0046	0.0769	0.3191	0.5987	0.0754		0.7711
**X8isop**	0.2413	0.1892	0.8131	0.4985	0.9570	0.0396	0.8214	0.7711	
**TAS**	0.3957	0.9723	0.9701	0.9871	0.5796	0.0013	0.8622	0.3808	0.0654
**TOS**	0.1220	0.5377	0.0844	0.0288	0.0679	0.0981	0.5811	0.1113	0.3050
**OSI**	0.0807	0.4709	0.0035	0.0699	0.1179	0.6413	0.8489	0.0776	0.7474
**Px**	0.3100	0.9991	0.5457	0.9289	0.8860	0.0000	0.0781	0.9793	0.0149
**AGE**	0.2055	0.9353	0.6217	0.9191	0.9311	0.0000	0.0305	0.9121	0.0159
**CAT**	0.2415	0.9163	0.0265	0.4629	0.5557	0.8219	0.8473	0.5124	0.4261
**pPh**	0.8978	0.8019	0.9299	0.4978	0.5164	0.6622	0.3470	0.9612	0.7199

P

	**TAS**	**TOS**	**OSI**	**Px**	**AGE**	**CAT**	**pPh**
**UWS**	0.3957	0.1220	0.0807	0.3100	0.2055	0.2415	0.8978
**DMFT**	0.9723	0.5377	0.4709	0.9991	0.9353	0.9163	0.8019
**PI**	0.9701	0.0844	0.0035	0.5457	0.6217	0.0265	0.9299
**pH**	0.9871	0.0288	0.0699	0.9289	0.9191	0.4629	0.4978
**SBC**	0.5796	0.0679	0.1179	0.8860	0.9311	0.5557	0.5164
**TP**	0.0013	0.0981	0.6413	0.0000	0.0000	0.8219	0.6622
**MDA**	0.8622	0.5811	0.8489	0.0781	0.0305	0.8473	0.3470
**AOPP**	0.3808	0.1113	0.0776	0.9793	0.9121	0.5124	0.9612
**X8isop**	0.0654	0.3050	0.7474	0.0149	0.0159	0.4261	0.7199
**TAS**		0.2348	0.6062	0.0039	0.0059	0.1810	0.1453
**TOS**	0.2348		0.0000	0.0400	0.0561	0.9718	0.0148
**OSI**	0.6062	0.0000		0.3904	0.3356	0.1644	0.9153
**Px**	0.0039	0.0400	0.3904		0.0000	0.6560	0.4916
**AGE**	0.0059	0.0561	0.3356	0.0000		0.5587	0.7269
**CAT**	0.1810	0.9718	0.1644	0.6560	0.5587		0.7269
**pPh**	0.1453	0.0148	0.9153	0.4916	0.7269	0.2578	

PC, patients with breast cancer and with the BRCA1 mutation; AOPP, advanced oxidation protein products, TP; total protein; OSI, oxidative stress index; TOS, total oxidant status; TAS, total antioxidant status, CAT, Catalase activity; pPh, total phenolic content, Pl.I score, Plaque Index; DMFT, decay, missing, filled teeth; UWS, unstimulated whole saliva; SBC, salivary buffer capacity; 8-isop, concentration of 8-isoprostane, MDA, concentration of malondialdehyde; AGE, advanced glycation end-products; SBC, buffer capacity of saliva, Px, activity of salivary peroxidase.

Table S2. R and *p*-value of correlation between parameters measured in the saliva of women with the BRCA1 mutation (PZ).

R											
	**UWS**	**DMFT**	**PI**	**pH**	**SBC**	**TP**	**MDA**	**AOPP**	**X8isop**	**TAS**	**TOS**
**UWS**	1.00	−0.42	−0.53	0.18	0.35	−0.23	0.40	0.26	0.07	0.10	0.25
**DMFT**	−0.42	1.00	0.52	−0.33	−0.11	−0.33	0.06	0.07	0.08	0.36	−0.38
**PI**	−0.53	0.52	1.00	−0.09	−0.09	0.01	−0.41	−0.16	−0.10	0.11	−0.33
**pH**	0.18	−0.33	−0.09	1.00	0.29	−0.23	0.37	0.06	0.46	0.02	0.32
**SBC**	0.35	−0.11	−0.09	0.29	1.00	0.18	0.17	−0.08	0.20	0.25	0.41
**TP**	−0.23	−0.33	0.01	−0.23	0.18	1.00	−0.74	−0.58	−0.49	−0.53	−0.05
**MDA**	0.40	0.06	−0.41	0.37	0.17	−0.74	1.00	0.57	0.42	0.42	0.22
**AOPP**	0.26	0.07	−0.16	0.06	−0.08	−0.58	0.57	1.00	0.06	0.28	0.10
**X8isop**	0.07	0.08	−0.10	0.46	0.20	−0.49	0.42	0.06	1.00	0.48	0.51
**TAS**	0.10	0.36	0.11	0.02	0.25	−0.53	0.42	0.28	0.48	1.00	0.17
**TOS**	0.25	−0.38	−0.33	0.32	0.41	−0.05	0.22	0.10	0.51	0.17	1.00
**OSI**	0.17	−0.51	−0.29	0.15	0.37	0.39	−0.09	−0.11	0.11	−0.21	0.81
**Px**	0.35	0.11	−0.25	0.30	0.11	−0.76	0.75	0.58	0.54	0.54	0.50
**AGE**	0.24	0.29	−0.09	0.19	−0.06	−0.82	0.64	0.38	0.57	0.62	0.36
**CAT**	0.17	0.04	−0.15	0.11	−0.04	−0.44	0.14	0.12	0.49	0.35	0.33
**pPh**	−0.46	0.48	0.34	−0.39	−0.43	−0.11	−0.16	−0.12	0.12	0.26	−0.36

	**OSI**	**Px**	**AGE**	**CAT**	**pPh**
**UWS**	0.17	0.35	0.24	0.17	−0.46
**DMFT**	−0.51	0.11	0.29	0.04	0.48
**PI**	−0.29	−0.25	−0.09	−0.15	0.34
**pH**	0.15	0.30	0.19	0.11	−0.39
**SBC**	0.37	0.11	−0.06	−0.04	−0.43
**TP**	0.39	−0.76	−0.82	−0.44	−0.11
**MDA**	−0.09	0.75	0.64	0.14	−0.16
**AOPP**	−0.11	0.58	0.38	0.12	−0.12
**X8isop**	0.11	0.54	0.57	0.49	0.12
**TAS**	−0.21	0.54	0.62	0.35	0.26
**TOS**	0.81	0.50	0.36	0.33	−0.36
**OSI**	1.00	0.16	−0.04	0.00	−0.35
**Px**	0.16	1.00	0.90	0.39	−0.03
**AGE**	−0.04	0.90	1.00	0.53	0.14
**CAT**	0.00	0.39	0.53	1.00	0.16
**pPh**	−0.35	−0.03	0.14	0.16	1.00
##					n = 20

P									
	**UWS**	**DMFT**	**PI**	**pH**	**SBC**	**TP**	**MDA**	**AOPP**	**X8isop**
**UWS**		0.0517	0.0109	0.4139	0.1145	0.3036	0.0679	0.2496	0.7572
**DMFT**	0.0517		0.0137	0.1320	0.6390	0.1364	0.7752	0.7672	0.7256
**PI**	0.0109	0.0137		0.6988	0.6743	0.9640	0.0590	0.4895	0.6620
**pH**	0.4139	0.1320	0.6988		0.1862	0.3041	0.0931	0.7961	0.0315
**SBC**	0.1145	0.6390	0.6743	0.1862		0.4115	0.4440	0.7233	0.3841
**TP**	0.3036	0.1364	0.9640	0.3041	0.4115		0.0000	0.0043	0.0197
**MDA**	0.0679	0.7752	0.0590	0.0931	0.4440	0.0000		0.0059	0.0513
**AOPP**	0.2496	0.7672	0.4895	0.7961	0.7233	0.0043	0.0059		0.7907
**X8isop**	0.7572	0.7256	0.6620	0.0315	0.3841	0.0197	0.0513	0.7907	
**TAS**	0.6649	0.0959	0.6302	0.9442	0.2626	0.0108	0.0533	0.2117	0.0234

**TOS**	0.2653	0.0822	0.1357	0.1474	0.0579	0.8333	0.3232	0.6507	0.0153
**OSI**	0.4365	0.0154	0.1958	0.5027	0.0856	0.0703	0.6918	0.6131	0.6414
**Px**	0.1089	0.6138	0.2715	0.1790	0.6345	0.0000	0.0000	0.0051	0.0089
**AGE**	0.2806	0.1948	0.7007	0.3857	0.7970	0.0000	0.0014	0.0815	0.0058
**CAT**	0.4484	0.8476	0.5097	0.6355	0.8583	0.0428	0.5453	0.5866	0.0214
**pPh**	0.0324	0.0224	0.1181	0.0765	0.0445	0.6117	0.4749	0.6099	0.5913

			**P**				
	**TAS**	**TOS**	**OSI**	**Px**	**AGE**	**CAT**	**pPh**
**UWS**	0.6649	0.2653	0.4365	0.1089	0.2806	0.4484	0.0324
**DMFT**	0.0959	0.0822	0.0154	0.6138	0.1948	0.8476	0.0224
**PI**	0.6302	0.1357	0.1958	0.2715	0.7007	0.5097	0.1181
**pH**	0.9442	0.1474	0.5027	0.1790	0.3857	0.6355	0.0765
**SBC**	0.2626	0.0579	0.0856	0.6345	0.7970	0.8583	0.0445
**TP**	0.0108	0.8333	0.0703	0.0000	0.0000	0.0428	0.6117
**MDA**	0.0533	0.3232	0.6918	0.0000	0.0014	0.5453	0.4749
**AOPP**	0.2117	0.6507	0.6131	0.0051	0.0815	0.5866	0.6099
**X8isop**	0.0234	0.0153	0.6414	0.0089	0.0058	0.0214	0.5913
**TAS**		0.4575	0.3390	0.0091	0.0019	0.1078	0.2428
**TOS**	0.4575		0.0000	0.0190	0.1031	0.1338	0.1039
**OSI**	0.3390	0.0000		0.4636	0.8567	0.9931	0.1140
**Px**	0.0091	0.0190	0.4636		0.0000	0.0712	0.8915
**AGE**	0.0019	0.1031	0.8567	0.0000		0.0105	0.5316
**CAT**	0.1078	0.1338	0.9931	0.0712	0.0105		0.4676
**pPh**	0.2428	0.1039	0.1140	0.8915	0.5316	0.4676	

PZ, generally healthy patients with the BRCA1 mutation; AOPP, advanced oxidation protein products, TP; total protein; OSI, oxidative stress index; TOS, total oxidant status; TAS, total antioxidant status, CAT, Catalase activity; pPh, total phenolic content, Pl.I score, Plaque Index; DMFT, decay, missing, filled teeth; UWS, unstimulated whole saliva; SBC, salivary buffer capacity; 8-isop, concentration of 8-isoprostane, MDA, concentration of malondialdehyde; AGE, advanced glycation end-products; SBC, buffer capacity of saliva, Px, activity of salivary peroxidase.

Table S3. R and *p*-value of correlation between parameters measured in the saliva of healthy women without the BRCA1 mutation (NZ).

	R										
	**UWS**	**DMFT**	**PI**	**pH**	**SBC**	**TP**	**MDA**	**AOPP**	**X8isop**	**TAS**	**TOS**
**UWS**	1.00	−0.09	−0.37	−0.05	0.17	−0.30	−0.09	0.04	−0.27	0.28	−0.43
**DMFT**	−0.09	1.00	0.29	−0.37	−0.36	−0.04	−0.20	0.06	0.12	−0.13	0.17
**PI**	−0.37	0.29	1.00	−0.27	−0.14	0.00	0.30	0.05	0.52	0.07	0.34
**pH**	−0.05	−0.37	−0.27	1.00	0.37	−0.11	0.13	−0.38	−0.15	0.30	0.17
**SBC**	0.17	−0.36	−0.14	0.37	1.00	−0.15	0.30	−0.21	−0.07	0.20	−0.14
**TP**	−0.30	−0.04	0.00	−0.11	−0.15	1.00	−0.48	0.24	−0.24	−0.78	0.26
**MDA**	−0.09	−0.20	0.30	0.13	0.30	−0.48	1.00	−0.07	0.74	0.46	0.13
**AOPP**	0.04	0.06	0.05	−0.38	−0.21	0.24	−0.07	1.00	0.18	−0.37	0.09
**X8isop**	−0.27	0.12	0.52	−0.15	−0.07	−0.24	0.74	0.18	1.00	0.21	0.16
**TAS**	0.28	−0.13	0.07	0.30	0.20	−0.78	0.46	−0.37	0.21	1.00	−0.14
**TOS**	−0.43	0.17	0.34	0.17	−0.14	0.26	0.13	0.09	0.16	−0.14	1.00
**OSI**	−0.40	0.10	0.34	0.12	−0.15	0.47	0.00	0.21	0.12	v0.28	0.95
**Px**	0.40	0.07	−0.07	0.19	0.09	−0.86	0.45	−0.02	0.25	0.74	−0.18
**AGE**	0.14	0.01	0.09	0.19	0.28	−0.80	0.67	−0.18	0.52	0.76	−0.16
**CAT**	0.03	0.10	0.31	0.23	−0.12	−0.22	0.15	−0.01	0.16	0.21	0.26
**pPh**	−0.04	−0.19	0.00	0.23	0.03	−0.25	0.40	−0.02	0.50	0.25	−0.18

	**OSI**	**Px**	**AGE**	**CAT**	**pPh**
**UWS**	−0.40	0.40	0.14	0.03	−0.04
**DMFT**	0.10	0.07	0.01	0.10	−0.19
**PI**	0.34	−0.07	0.09	0.31	0.00
**pH**	0.12	0.19	0.19	0.23	0.23
**SBC**	0.15	0.09	0.28	-0.12	0.03
**TP**	0.47	−0.86	−0.80	-0.22	−0.25
**MDA**	0.00	0.45	0.67	0.15	0.40
**AOPP**	0.21	−0.02	−0.18	-0.01	−0.02
**X8isop**	0.12	0.25	0.52	0.16	0.50
**TAS**	−0.28	0.74	0.76	0.21	0.25
**TOS**	0.95	−0.18	−0.16	0.26	−0.18
**OSI**	1.00	−0.33	−0.29	0.21	−0.22
**Px**	−0.33	1.00	0.72	0.47	0.28
**AGE**	−0.29	0.72	1.00	0.15	0.47
**CAT**	0.21	0.47	0.15	1.00	0.22
**pPh**	−0.22	0.28	0.47	0.22	1.00
					n = 25

	P								
	**UWS**	**DMFT**	**PI**	**pH**	**SBC**	**TP**	**MDA**	**AOPP**	**X8isop**
**UWS**		0.6584	0.0677	0.8105	0.4103	0.1418	0.6690	0.8504	0.1933
**DMFT**	0.6584		0.1575	0.0655	0.0741	0.8426	0.3444	0.7897	0.5728
**PI**	0.0677	0.1575		0.1914	0.5034	0.9868	0.1459	0.7993	0.0081
**pH**	0.8105	0.0655	0.1914		0.0688	0.6028	0.5488	0.0647	0.4679
**SBC**	0.4103	0.0741	0.5034	0.0688		0.4745	0.1467	0.3044	0.7474
**TP**	0.1418	0.8426	0.9868	0.6028	0.4745		0.0153	0.2453	0.2469
**MDA**	0.6690	0.3444	0.1459	0.5488	0.1467	0.0153		0.7421	0.0000
**AOPP**	0.8504	0.7897	0.7993	0.0647	0.3044	0.2453	0.7421		0.3864
**X8isop**	0.1933	0.5728	0.0081	0.4679	0.7474	0.2469	0.0000	0.3864	
**TAS**	0.1717	0.5336	0.7476	0.1382	0.3304	0.0000	0.0206	0.0659	0.3181

**TOS**	0.0325	0.4271	0.0948	0.4296	0.5131	0.2007	0.5452	0.6786	0.4332
**OSI**	0.0448	0.6195	0.0960	0.5837	0.4863	0.0189	0.9875	0.3100	0.5829
**Px**	0.0463	0.7312	0.7442	0.3605	0.6796	0.0000	0.0231	0.9338	0.2186
**AGE**	0.4930	0.9595	0.6809	0.3508	0.1746	0.0000	0.0002	0.3897	0.0077
**CAT**	0.9027	0.6292	0.1327	0.2757	0.5737	0.2807	0.4878	0.9744	0.4434
**pPh**	0.8617	0.3754	0.9838	0.2692	0.9026	0.2238	0.0484	0.9312	0.0109

	**TAS**	**TOS**	**OSI**	**Px**	**AGE**	**CAT**	**pPh**
**UWS**	0.1717	0.0325	0.0448	0.0463	0.4930	0.9027	0.8617
**DMFT**	0.5336	0.4271	0.6195	0.7312	0.9595	0.6292	0.3754
**PI**	0.7476	0.0948	0.0960	0.7442	0.6809	0.1327	0.9838
**pH**	0.1382	0.4296	0.5837	0.3605	0.3508	0.2757	0.2692
**SBC**	0.3304	0.5131	0.4863	0.6796	0.1746	0.5737	0.9026
**TP**	0.0000	0.2007	0.0189	0.0000	0.0000	0.2807	0.2238
**MDA**	0.0206	0.5452	0.9875	0.0231	0.0002	0.4878	0.0484
**AOPP**	0.0659	0.6786	0.3100	0.9338	0.3897	0.9744	0.9312
**X8isop**	0.3181	0.4332	0.5829	0.2186	0.0077	0.4434	0.0109
**TAS**		0.5153	0.1684	0.0000	0.0000	0.3246	0.2334
**TOS**	0.5153		0.0000	0.3799	0.4462	0.2114	0.3894
**OSI**	0.1684	0.0000		0.1112	0.1672	0.3218	0.2830
**Px**	0.0000	0.3799	0.1112		0.0000	0.0178	0.1788
**AGE**	0.0000	0.4462	0.1672	0.0000		0.4852	0.0183
**CAT**	0.3246	0.2114	0.3218	0.0178	0.4852		0.2893
**pPh**	0.2334	0.3894	0.2830	0.1788	0.0183	0.2893a	

NZ, generally healthy patients without the BRCA1 mutation; AOPP, advanced oxidation protein products, TP; total protein; OSI, oxidative stress index; TOS,total oxidant status; TAS, total antioxidant status, CAT, Catalase activity; pPh, total phenolic content, Pl.I score, Plaque Index; DMFT, decay, missing, filled teeth; UWS, unstimulated whole saliva; SBC, salivary buffer capacity; 8-isop, concentration of 8-isoprostane, MDA, concentration of malondialdehyde; AGE, advanced glycation end-products; SBC, buffer capacity of saliva, Px, activity of salivary peroxidase.

Table S4. R and *p*-value of correlation between parameters analysed in the saliva of women with breast cancer and without the BRCA1 mutation (NC).

R											
**TAB**	**UWS**	**DMFT**	**PI**	**pH**	**SBC**	**TP**	**MDA**	**AOPP**	**X8isop**	**TAS**	**TOS**
**UWS**	1.00	0.37	0.26	0.02	0.15	−0.22	−0.10	−0.34	0.66	0.09	−0.24
**DMFT**	0.37	1.00	0.00	−0.77	0.68	0.25	−0.27	−0.36	0.38	−0.09	−0.45
**PI**	0.26	0.00	1.00	0.26	−0.38	−0.07	0.10	−0.38	0.30	−0.11	0.31
**pH**	0.02	−0.77	0.26	1.00	−0.40	0.06	0.12	−0.12	−0.20	−0.20	0.25
**SBC**	0.15	0.68	−0.38	−0.40	1.00	0.56	−0.38	−0.25	−0.08	−0.47	−0.38
**TP**	−0.22	0.25	−0.07	0.06	0.56	1.00	−0.05	−0.52	−0.12	−0.87	−0.34
**MDA**	−0.10	−0.27	0.10	0.12	−0.38	−0.05	1.00	−0.42	−0.38	−0.10	−0.43
**AOPP**	−0.34	−0.36	−0.38	−0.12	−0.25	−0.52	−0.42	1.00	−0.06	0.48	0.70
**X8isop**	0.66	0.38	0.30	−0.20	−0.08	−0.12	−0.38	−0.06	1.00	0.16	−0.01
**TAS**	0.09	−0.09	−0.11	−0.20	−0.47	−0.87	−0.10	0.48	0.16	1.00	0.15
**TOS**	−0.24	−0.45	0.31	0.25	−0.38	−0.34	−0.43	0.70	−0.01	0.15	1.00
**OSI**	−0.20	−0.43	0.28	0.36	−0.15	−0.01	−0.40	0.52	−0.04	−0.26	0.91
**Px**	−0.31	−0.37	−0.09	−0.13	−0.64	−0.84	0.08	0.66	−0.15	0.87	0.39
**AGE**	0.40	0.01	−0.05	−0.15	−0.30	−0.86	−0.25	0.40	0.35	0.92	0.12
**CAT**	0.65	0.22	0.13	−0.07	0.09	−0.27	−0.70	0.30	0.80	0.21	0.33
**pPh**	−0.01	0.31	0.45	0.12	0.41	0.75	−0.18	−0.60	0.09	−0.72	−0.13

	**OSI**	**Px**	**AGE**	**CAT**	**pPh**
**UWS**	−0.20	−0.31	0.40	0.65	−0.01
**DMFT**	−0.43	−0.37	0.01	0.22	0.31
**PI**	0.28	−0.09	−0.05	0.13	0.45
**pH**	0.36	−0.13	−0.15	−0.07	0.12
**SBC**	−0.15	−0.64	−0.30	0.09	0.41
**TP**	−0.01	−0.84	−0.86	−0.27	0.75
**MDA**	−0.40	0.08	−0.25	−0.70	−0.18
**AOPP**	0.52	0.66	0.40	0.30	−0.60
**X8isop**	−0.04	−0.15	0.35	0.80	0.09
**TAS**	−0.26	0.87	0.92	0.21	−0.72
**TOS**	0.91	0.39	0.12	0.33	−0.13
**OSI**	1.00	0.01	−0.21	0.31	0.13
**Px**	0.01	1.00	0.67	−0.07	−0.74
**AGE**	−0.21	0.67	1.00	0.50	−0.59
**CAT**	0.31	−0.07	0.50	1.00	−0.01
**pPh**	0.13	−0.74	−0.59	−0.01	1.00
					n = 9

P									
	**UWS**	**DMFT**	**PI**	**pH**	**SBC**	**TP**	**MDA**	**AOPP**	**X8isop**
**UWS**		0.3293	0.4926	0.9626	0.6944	0.5669	0.7890	0.3659	0.0513
**DMFT**	0.3293		0.9945	0.0151	0.0431	0.5087	0.4857	0.3361	0.3152
**PI**	0.4926	0.9945		0.4981	0.3180	0.8655	0.7991	0.3133	0.4390
**pH**	0.9626	0.0151	0.4981		0.2880	0.8762	0.7507	0.7525	0.6094
**SBC**	0.6944	0.0431	0.3180	0.2880		0.1173	0.3113	0.5125	0.8376
**TP**	0.5669	0.5087	0.8655	0.8762	0.1173	a	0.8917	0.1499	0.7646
**MDA**	0.7890	0.4857	0.7991	0.7507	0.3113	0.8917	a	0.2565	0.3194
**AOPP**	0.3659	0.3361	0.3133	0.7525	0.5125	0.1499	0.2565	a	0.8790
**X8isop**	0.0513	0.3152	0.4390	0.6094	0.8376	0.7646	0.3194	0.8790	a
**TAS**	0.8256	0.8207	0.7866	0.6097	0.2058	0.0021	0.7907	0.1907	0.6901

**TOS**	0.5394	0.2233	0.4224	0.5165	0.3166	0.3677	0.2470	0.0358	0.9883
**OSI**	0.6055	0.2425	0.4678	0.3472	0.6962	0.9810	0.2840	0.1503	0.9122
**Px**	0.4208	0.3275	0.8224	0.7392	0.0619	0.0045	0.8435	0.0523	0.6956
**AGE**	0.2913	0.9731	0.8888	0.7027	0.4358	0.0026	0.5116	0.2852	0.3557
**CAT**	0.0579	0.5639	0.7389	0.8649	0.8161	0.4784	0.0350	0.4403	0.0089
**pPh**	0.9817	0.4220	0.2275	0.7613	0.2678	0.0189	0.6347	0.0878	0.8130

P

Tab	TAS	TOS	OSI	Px	AGE	CAT	pPh
**UWS**	0.8256	0.5394	0.6055	0.4208	0.2913	0.0579	0.9817
**DMFT**	0.8207	0.2233	0.2425	0.3275	0.9731	0.5639	0.4220
**PI**	0.7866	0.4224	0.4678	0.8224	0.8888	0.7389	0.2275
**pH**	0.6097	0.5165	0.3472	0.7392	0.7027	0.8649	0.7613
**SBC**	0.2058	0.3166	0.6962	0.0619	0.4358	0.8161	0.2678
**TP**	0.0021	0.3677	0.9810	0.0045	0.0026	0.4784	0.0189
**MDA**	0.7907	0.2470	0.2840	0.8435	0.5116	0.0350	0.6347
**AOPP**	0.1907	0.0358	0.1503	0.0523	0.2852	0.4403	0.0878
**X8isop**	0.6901	0.9883	0.9122	0.6956	0.3557	0.0089	0.8130
**TAS**		0.7008	0.5046	0.0024	0.0005	0.5845	0.0292
**TOS**	0.7008		0.0008	0.2944	0.7553	0.3843	0.7410
**OSI**	0.5046	0.0008		0.9739	0.5943	0.4166	0.7306
**Px**	0.0024	0.2944	0.9739		0.0498	0.8611	0.0213
**AGE**	0.0005	0.7553	0.5943	0.0498		0.1701	0.0954
**CAT**	0.5845	0.3843	0.4166	0.8611	0.1701		0.9808
**pPh**	0.0292	0.7410	0.7306	0.0213	0.0954	0.9808	

NC, patients with breast cancer but without the BRCA1 mutation, AOPP advanced oxidation protein products, TP; total protein; OSI, oxidative stress index; TOS, total oxidant status; TAS, total antioxidant status, CAT, Catalase activity; pPh, total phenolic content, Pl.I score, Plaque Index; DMFT, decay, missing, filled teeth; UWS, unstimulated whole saliva; SBC, salivary buffer capacity; 8-isop, concentration of 8-isoprostane, MDA, concentration of malondialdehyde; AGE, advanced glycation end-products; SBC, buffer capacity of saliva, Px, activity of salivary peroxidase.
